# PLENTY, a hydroxyproline *O*-arabinosyltransferase, negatively regulates root nodule symbiosis in *Lotus japonicus*

**DOI:** 10.1093/jxb/ery364

**Published:** 2018-10-23

**Authors:** Emiko Yoro, Hanna Nishida, Mari Ogawa-Ohnishi, Chie Yoshida, Takuya Suzaki, Yoshikatsu Matsubayashi, Masayoshi Kawaguchi

**Affiliations:** 1 Division of Symbiotic Systems, National Institute for Basic Biology, Okazaki, Aichi, Japan; 2 Department of Basic Biology, School of Life Science, Graduate University for Advanced Studies (SOKENDAI), Okazaki, Aichi, Japan; 3 Graduate School of Life and Environmental Sciences, University of Tsukuba, Tsukuba, Ibaraki, Japan; 4 Division of Biological Science, Graduate School of Science, Nagoya University Chikusa, Nagoya, Japan

**Keywords:** Arabinosyltransferase, CLE peptide, glycosylation, legume–rhizobial symbiosis, *Lotus japonicus*, nodulation control, post-translational modification

## Abstract

Legumes can survive in nitrogen-deficient environments by forming root-nodule symbioses with rhizobial bacteria; however, forming nodules consumes energy, and nodule numbers must thus be strictly controlled. Previous studies identified major negative regulators of nodulation in *Lotus japonicus*, including the small peptides CLAVATA3/ESR (CLE)-RELATED-ROOT SIGNAL1 (CLE-RS1), CLE-RS2, and CLE-RS3, and their putative major receptor HYPERNODULATION AND ABERRANT ROOT FORMATION1 (HAR1). *CLE-RS2* is known to be expressed in rhizobia-inoculated roots, and is predicted to be post-translationally arabinosylated, a modification essential for its activity. Moreover, all three CLE-RSs suppress nodulation in a HAR1-dependent manner. Here, we identified *PLENTY* as a gene responsible for the previously isolated hypernodulation mutant *plenty*. *PLENTY* encoded a hydroxyproline *O*-arabinosyltransferase orthologous to *ROOT DETERMINED NODULATION1* in *Medicago truncatula*. PLENTY was localized to the Golgi, and an *in vitro* analysis of the recombinant protein demonstrated its arabinosylation activity, indicating that CLE-RS1/2/3 may be substrates for PLENTY. The constitutive expression experiments showed that CLE-RS3 was the major candidate substrate for PLENTY, suggesting the substrate preference of PLENTY for individual CLE-RS peptides. Furthermore, a genetic analysis of the *plenty har1* double mutant indicated the existence of another PLENTY-dependent and HAR1-independent pathway negatively regulating nodulation.

## Teaser

The gene responsible for the *plenty* hypernodulation phenotype in *Lotus japonicus* was identified. The enzymatic activity and Golgi localization of PLENTY demonstrated its function as a post-translational modification enzyme.

## Introduction

Legumes have evolved the ability to make specialized root organs called nodules, in which nitrogen-fixing symbionts, rhizobial bacteria, reside. This symbiosis enables legumes to grow on nitrogen-limited soils; however, the beneficial nitrogen acquisition is balanced against the high energy inputs required to fuel cell division in the nodules, as well as the allocation of photoassimilates to the rhizobia (Udvardi and Poole, 2013). Legumes have therefore developed mechanisms for maintaining the symbiotic balance between forming nodules to satisfy their nitrogen requirements and sustaining the energy levels required for other biological processes; these mechanisms are termed the autoregulation of nodulation (AON) (Kosslak and Bohlool, 1984; Caetano-Anollés and Gresshoff, 1990, 1991). The potential AON mechanisms involving systemic signaling between the root and shoot were originally determined using split-root and grafting techniques (Kosslak and Bohlool, 1984; Delves *et al.*, 1986), and the molecules participating in the negative control of nodulation have been elucidated (reviewed by (Magori and Kawaguchi, 2009; Reid *et al.*, 2011*b*; Suzaki *et al.*, 2015).

The current plausible model of AON via a systemic root-derived signal involves the expression of the systemic negative regulators, such as *CLAVATA3/ESR* (*CLE*)*-RELATED-ROOT SIGNAL1* (*CLE-RS1*), *CLE-RS2*, and *CLE-RS3* in *Lotus japonicus*, directly induced by transcription factors, NODULE INCEPTION (NIN) or NIN-like protein NITRATE UNRESPONSIVE SYMBIOSIS1 (NRSYM1) (Soyano *et al.*, 2014; Nishida *et al.*, 2016; Nishida and Suzaki, 2018; Nishida *et al.*, 2018). Other related CLE peptides negatively regulating nodulation have also been found in *L. japonicu*s and other legumes, including *Medicago truncatula*, pea (*Pisum sativum*), and soybean (*Glycine max*) (Mortier *et al.*, 2010,

Other AON factors closely related to PLENTY, namely ROOT DETERMINED NODULATION1 (RDN1) and NODULATION3 (NOD3), were found in *M. truncatula* and in pea, respectively. MtRDN1 and PsNOD3 are orthologous and have been suggested as factors that function in the root but not in the shoot (Li *et al.*, 2009; Novák, 2010; Schnabel *et al.*, 2011; Osipova *et al.*, 2012; Kassaw *et al.*, 2017), but their molecular functions had been unclear. Prior to the functional identification of *MtRDN1/PsNOD3*, their Arabidopsis homologs were reported as hydroxyproline *O*-arabinosyltransferases (HPATs) and termed HPAT1 (At2g25260), HPAT2 (At5g13500), and HPAT3 (At5g25265), which have recently been classified into the GT95 glycosyltransferase family (Showalter and Basu, 2016). *At*HPATs redundantly contribute to transferring an l-arabinosyl residue to the hydroxyl group of the hydroxyproline residues of several substrates, including extensin, *At*CLE2, and Arabidopsis plant peptide containing sulfated tyrosine 1 (*At*PSY1) (Ogawa-Ohnishi *et al.*, 2013; MacAlister *et al.*, 2016). This discovery raised the possibility that *Lj*PLENTY, *Mt*RDN1, or *Ps*NOD3 mediates the arabinosylation of their CLE peptides functioning in AON in the respective species. Additionally, a recent study of the *Mt*RDNs reported that, of the two CLE peptides, only *Mt*CLE12 is required for a functional *Mt*RDN1 to repress nodulation, suggesting that *Mt*RDN1 post-translationally modifies *Mt*CLE12 but not *Mt*CLE13 (Kassaw *et al.*, 2017).

The *plenty* mutant was previously isolated as a hypernodulator, but the gene responsible for this phenotype has not yet been identified (Yoshida *et al.*, 2010). Here, we identify the *plenty* locus and characterize *PLENTY* as an ortholog of *MtRDN1* and *PsNOD3* (Postma *et al.*, 1988; Sagan and Duc, 1996; Schnabel *et al.*, 2011). We determine the localization of PLENTY to the Golgi and purified recombinant PLENTY protein to detect its HPAT activity. This biochemical assay using an artificially synthesized peptide as a substrate for arabinosylation provided direct molecular evidence that PLENTY acts as a post-translational modification HPAT enzyme. Furthermor e, the constitutive expression of *CLE-RS1/2/3* in *plenty* suggested that PLENTY preferentially associates with CLE-RS3 peptides as substrate. Finally, *plenty har1* double mutant analysis indicates the existence of an unknown substrate for PLENTY other than CLE-RS1/2/3, which is not accepted by HAR1. These findings provide a molecular clue for understanding how PLENTY regulates nodulation.

## Materials and methods

### Growth conditions

*Lotus japonicus* (Miyakojima MG-20) seeds were sown in sterilized vermiculite, to which Broughton and Dilworth (B&D) solution (Broughton and Dilworth, 1971) containing 0.5 mM KNO_3_ . Seeds were inoculated with *Mesorhizobium loti* MAFF303099, while the control seedlings were not inoculated. Seedlings were grown under 16 h light/8 h dark cycles at 24 °C.

### Map-based cloning, genomic PCR, and 5'-/3'-RACE

The *plenty* mutants were backcrossed three times to the parental plant ‘Miyakojima MG-20’ and crossed with another accession, ‘Gifu B-129’. DNA polymorphisms in the genomes of the 1087 F_2_ progeny displaying the *plenty* mutant phenotype were identified using a series of genetic markers (http://www.kazusa.or.jp/lotus/markerdb_index.html, last accessed 25 October 2018) and two genetic markers newly developed in this study, EY004 and EY005 (see Supplementary Table S1 at *JXB* online). Genomic DNA was extracted with a DNeasy Plant Mini Kit (Qiagen). The ~16 kb deletion in the *plenty* mutant was identified using genomic PCR (Supplementary Fig. S1), with the sets of primers shown in Supplementary Table S2. The 5' and 3' ends of *PLENTY*, *PLENTY2*, and *PLENTY3* were determined using a SMARTer^®^ RACE cDNA Amplification Kit (Clontech), utilizing RNA extracted from whole roots inoculated with *M. loti* MAFF303099. The DDBJ accession numbers for the *PLENTY*, *PLENTY2*, and *PLENTY3* mRNAs were LC010646–LC010648 (http://getentry.ddbj.nig.ac.jp/top-e.html, last accessed 25 October 2018).

### Plasmid construction

A deletion series of PLENTY coding sequence (CDS), as well as a full-length CDS, were generated using reverse transcription and the appropriate primer sets (Supplementary Table S2), cloned into the pGEM^®^-T-Easy vector (Promega) using the TA strategy, and named pGEM-Full-PLENTY, pGEM-ΔN1-PLENTY, and pGEM-N-PLENTY. The full-length CDS clone starts at the seventh predicted codon of PLENTY compared with the previous report of *Mt*RDN1 (Schnabel *et al.*, 2011; Supplementary Appendix S2); at the time it was generated, that was the predicted start of the CDS. Next, each construct was digested with *Eco*RI and *Spe*I, inserted into the Gateway-based entry plasmid pJL-Blue (Suzaki *et al.*, 2012), and named pJL-Blue-Full-PLENTY, pJL-Blue-ΔN1-PLENTY, and pJL-Blue-N-PLENTY, respectively. For the complementation test, full-length *PLENTY* CDS fragments were inserted into the Gateway site of pUB-GW-HYG using an LR recombination reaction (Invitrogen) to generate pUB-GW-Full-PLENTY (Maekawa *et al.*, 2008). To produce PLENTY–green fluorescent protein (GFP) fusion proteins, the stop codon of each pJL-Blue-based entry vector was mutagenized using circular PCR with a phosphorylated set of primers (Supplementary Table S2), and the vector was then digested with *Dpn*I and self-ligated. The subcellular localization of PLENTY was analyzed using a series of *PLENTY* fragments from the mutagenized pJL-Blue-based entry vectors, which were inserted into pUGW5 for particle bombardment into onion (*Allium cepa*) epidermal cells and *L. japonicus* roots. The *PLENTY* fragments were also inserted into pGWB5 for the *Agrobacterium tumefaciens*-mediated infiltration of *Nicotiana benthamiana* using an LR recombination reaction (Invitrogen). The two constructs, pUGW5 and pGWB5 (Nakagawa *et al.*, 2007), were kindly provided by Dr Mano (National Institute for Basic Biology) and Dr Nakagawa (Shimane University), respectively. To detect the HPAT activities of the three *PLENTY* CDS fragments (full-length, ΔN1, and ΔN2), they were C-terminally fused to a FLAG tag using pGEM-Full-PLENTY as a template for the PCR (see Supplementary Table S2 for the list of primers). The fragments were inserted into pYES2 (Invitrogen) and linearized with *Bam*HI in an In-Fusion reaction (Takara). The plasmids used for the constitutive expression of *GUS* (β-glucuronidase), CLE3, *CLE-RS1*/*2* (Okamoto *et al.*, 2009) and *CLE-RS3* (Nishida *et al.*, 2016) were previously described.

### Hairy root and stable transformations of *L. japonicus*

Hairy root and stable transformations of *L. japonicus* were performed using the *Agrobacterium rhizogenes* strain AR1193 alone or in combination with the *Agrobacterium tumefaciens* strain AGL1 harboring the respective plasmids, as described previously (http://www.bio-protocol.org/wenzhang.aspx?id=795, http://www.bio-protocol.org/wenzhang.aspx?id=796, last accessed 25 October 2018). In the hairy root transformations constitutively expressing *GUS*, *CLE3*, and *CLE-RS1/2/3*, GFP fluorescence was used as a marker of transformation, and the successfully transformed roots were inoculated with *M. loti* MAFF303099. The nodule numbers and other phenotypes of the hairy roots and stably transformed roots were measured at 14 days after inoculation (DAI), while the growth phenotypes of the stable transformants were measured at 8 weeks after inoculation.

### Phylogenetic analysis

Forty-one amino acid sequences were obtained from the study of *MtRDN1* (Schnabel *et al.*, 2011) and an additional 16 amino acid sequences of *Brassica rapa* Chiifu-401 v1.2, *Carica papaya* ASGPB v0.4, *Gossypium raimondii* v2.1, and *Eucalyptus grandis* v2.0 genomes were obtained from a BLAST search for the Phytozome website (http://www.phytozome.net/, last accessed 25 October 2018). The total 57 amino acid sequences were aligned using MUSCLE (Edgar, 2004). All positions containing gaps and missing data were eliminated, and the final data set comprised a total of 266 amino acid positions. Evolutionary analyses were conducted in MEGA6 (Tamura *et al.*, 2013). The evolutionary history was inferred using the Maximum Likelihood method, based on the Le_Gascuel_2008 model (Le and Gascuel, 2008). The tree with the highest log likelihood (–7013.2051) is shown in Supplementary Fig. S2. The initial tree for the heuristic search was obtained by applying the Neighbor–Joining method to a matrix of pairwise distances estimated using the JTT model. A discrete Gamma distribution was used to model the evolutionary rate differences between the sites [five categories (+G, parameter=0.4454)].

### Subcellular localization analysis

Onion epidermal cells and *L. japonicus* roots of 3-day-old seedlings grown on moistened filter paper were transformed with each construct (pUGW5-based series) via particle bombardment with a Helios Gene Gun (BIO-RAD), as described previously (Mano *et al.*, 2006). To verify the co-localization of proteins with the Golgi network, *A. tumefaciens* AGL1 strains carrying each construct (pGWB5-based series) and an mCherry-fused Golgi marker construct (G-rb) (Nelson *et al.*, 2007) were mixed and co-infiltrated into *Nicotiana benthamiana* leaves using a p19-harboring strain, as previously described (Voinnet *et al.*, 2003; Kinoshita *et al.*, 2010).

### Microscopy observations

Bright-field and fluorescence images were generated using an SZX12/16 stereomicroscope or a BX50 microscope (Olympus) and a DP Controller (Olympus). Confocal images were generated using an A1 confocal laser-scanning microscope (Nikon) with a ×10 or ×20/0.75 NA objective lens.

### Detection of HPAT activity in PLENTY

PLENTY proteins were expressed as a C-terminal FLAG-tag fusion in the yeast (*Saccharomyces cerevisiae*) strain INVSc1, which was transformed with pYES2 vectors (Invitrogen) harboring full-length PLENTY or one of the two N-terminally deleted PLENTY constructs. Their expression was detected using immunoblotting, and the proteins were collected within a pellet of microsomal membrane, as described previously (Ogawa-Ohnishi *et al.*, 2013). HPAT activity assays were performed in 20 μl reaction mixtures containing 100 mM MOPS-KOH (pH 7.0) buffer, 1 mM MnCl_2_, 1.0% Triton X-100, 250 μM UDP-β-l-arabinofuranose (Peptide Institute, Inc.), 100 μM (PGVOOS)_3_ peptide, and 150 μg of total yeast microsomal membrane. The reaction mixture was incubated at 30 °C for 2 h, and then terminated by the addition of 100 μl of 0.1% formic acid. After centrifugation at 15000 rpm for 5 min, 40 µl aliquots of this solution were analyzed using LC/MS, as previously reported (Ogawa-Ohnishi *et al.*, 2013). The mass spectra were obtained by scanning the selected ion [(PGVOOS)_3_+Ala_1_: *m/z* 1849.6] in zoom scan mode.

### Double mutant analysis

To select the *plenty har1-7* homozygous double mutant, each plant was checked for the presence of an amplified polymorphic sequence marker in *har1-7* (a G1044A change in the *HAR1* CDS causing a W348 stop codon) (Magori *et al.*, 2009). Deletion of the *plenty* locus was detected using the primers listed in Supplementary Table S2. Nodules and other root phenotypes were counted and measured at 28 DAI.

### Gene expression analysis

Total RNA was isolated from each plant tissue at selected time points using an RNeasy Plant Mini Kit (Qiagen), and the first-strand cDNA was prepared using a QuantiTect Reverse Transcription Kit (Qiagen). Reverse transcription–quantitative PCR analysis was performed using an ABI Prism 7000 (Applied Biosystems) with THUNDERBIRD SYBR qPCR Mix (Toyobo) or with a QuanTitect SYBR Green RT-PCR Kit (Qiagen), according to the manufacturers’ protocols. *EF-1a* (GNf095a12) expression was used as the reference (Groth *et al.*, 2010). The relative expression levels were calculated using the ^ΔΔ^Ct method (Livak and Schmittgen, 2001). The primers used in the expression analysis are shown in Supplementary Table S2. The data are presented as the mean ±SD of three biological replicates or three technical replicates.

### Boxplot analysis

Boxplot analyses were performed in R using ggplot2 or basic R plotting commands. In the boxplots, the upper and lower ‘hinges’ correspond to the first and third quartiles. The upper whisker extends from the hinge to the highest value within 1.5× the interquartile range (IQR) between the first and third quartiles, while the lower whisker extends from the hinge to the lowest value within 1.5× IQR. Outliers (data beyond the end of the whiskers) are plotted as points.

## Results

### Identification of the *PLENTY* gene and phylogenetic analysis

The *plenty* mutant has two characteristic phenotypes, an increased number of nodules and short roots (Yoshida *et al.*, 2010). We previously reported that the *plenty* locus is located between markers TM0002 and TM0324 on the long arm of chromosome II in *L. japonicus*. We narrowed down the region of interest using map-based cloning utilizing a larger mapping population of 1087 F_2_ plants (Supplementary Fig. S1). A genomic PCR analysis of the region between marker TM0308 and the newly developed marker EY005 (Supplementary Table S1) revealed an ~16 kb deletion spanning two protein-coding genes, CM0308.590.r2.d/Lj2g3v3022950 encoding a putative telomerase-binding protein and CM0308.600.r2.d/Lj2g3v3022970 (http://www.kazusa.or.jp/lotus/). The latter gene, which we named *PLENTY*, was orthologous to *MtRDN1* (Supplementary Fig. S2; Schnabel *et al.*, 2011). Similar to *M. truncatula*, two other PLENTY paralogs termed *PLENTY2* and *PLENTY3*, were also identified in *L. japonicus*, and the three genes were phylogenetically divided into three related groups (Supplementary Fig. S2). There are no Arabidopsis homologs of *Lj*PLENTY and *Mt*RDN1; instead, there is a Brassicaceae-specific clade of *PLENTY*-like genes that are closely related to the group containing *LjPLENTY3* and *MtRDN3* (Schnabel *et al.*, 2011; Ogawa-Ohnishi *et al.*, 2013; Xu *et al.*, 2015). To explore further the Brassicaceae-specific evolution of the *PLENTY* genes, we performed a phylogenetic analysis using genes from *Brassica rapa*, *Carica papaya* (papaya), *Gossypium raimondii* (cotton), and *Eucalyptus grandis* (eucalyptus), acquired from the Phytozome database (http://www.phytozome.net/), as well as the same data set used in the study of *Mt*RDN1 (Schnabel *et al.*, 2011). Among these plants, all *PLENTY* homologs from the eudicots, except for those from *B. rapa*, were classified into groups 1–3.

### Nodule number and the non-symbiotic short-root phenotype of *plenty* were complemented by *PLENTY* expression

**Fig. 1.**
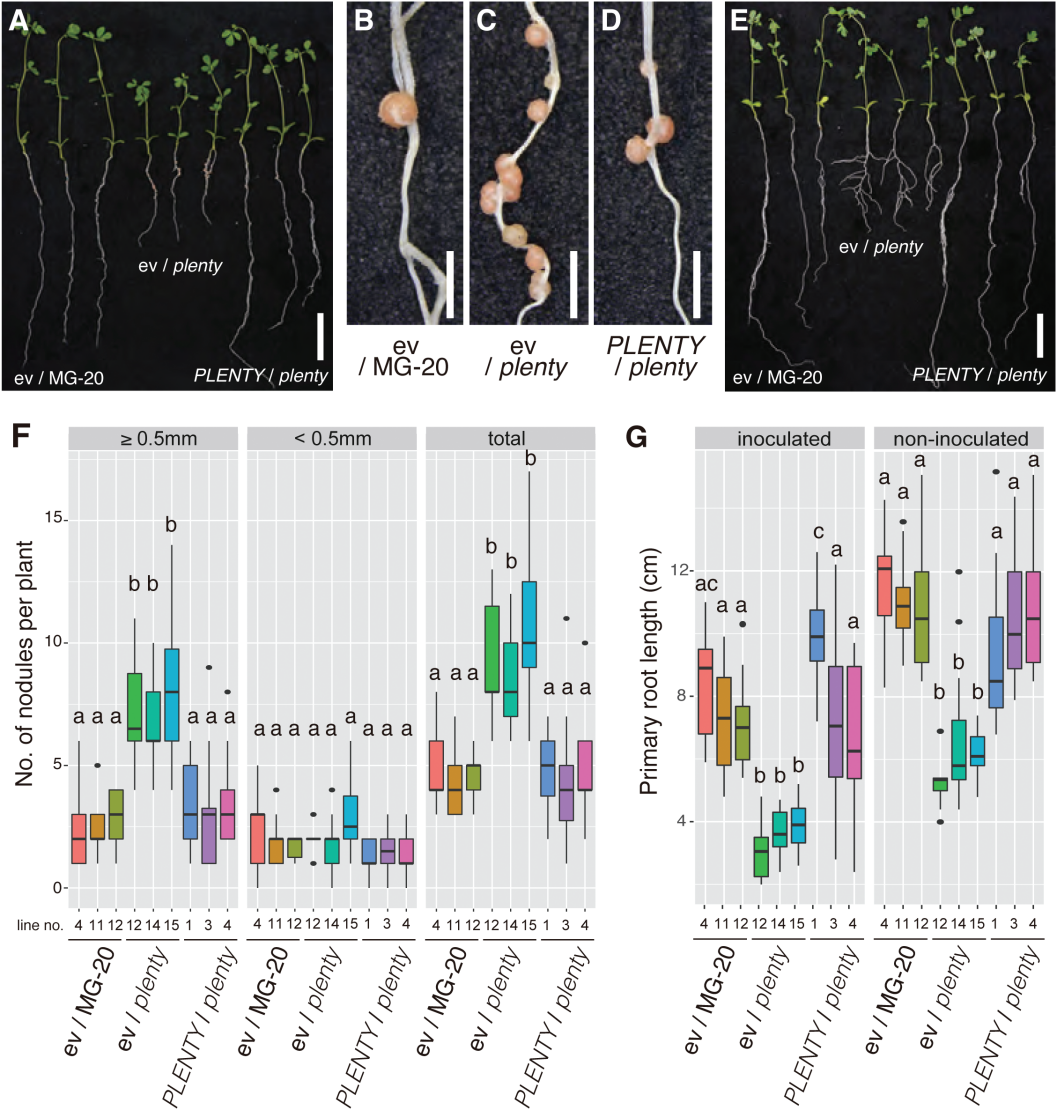
Complementation of *plenty*. (A) *Rhizobium*-inoculated MG-20 plants stably transformed with the empty vector pUB-GW-GFP (ev/MG-20), a *plenty* mutant transformed with the empty vector (ev/*plenty*), and a *plenty* mutant transformed with pUB-GW-Full-PLENTY (*PLENTY*/*plenty*) at 14 days after inoculation (DAI) with *M. loti* MAFF303099. Magnified images of the nodulated regions of the ev/MG-20 (B), ev/*plenty* (C), and *PLENTY*/*plenty* (D) plants are shown. (E) Non-inoculated plants of ev/MG-20, ev/*plenty*, and *PLENTY*/*plenty* at 21 days after germination (DAG). (F) Boxplots of the nodule numbers [≥0.5 mm diameter (left), <0.5 mm diameter (middle), and total (right)] of the individual inoculated T_3_ transgenic lines (*n*≥10). (G) Boxplots of the primary root length of the inoculated T_3_ transgenic plants at 14 DAI (left) and the non-inoculated T_3_ transgenic plant at 21 DAG (right). Scale bars=2 cm in (A, E) and 2 mm in (B–D). Different lower case letters represent statistically significant differences (*P*<0.05; Tukey’s HSD). Experiments were performed in triplicate (*n*≥10 in each trial).

The *plenty* mutant has an increased number of nodules and shorter roots in both symbiotic and non-symbiotic conditions (Yoshida *et al.*, 2010). We generated stably transformed *plenty* mutants harboring the *PLENTY* CDS under the control of the *LjUBIQUITIN* promoter or an empty vector (as the control) and observed their phenotypes in both inoculated and non-inoculated conditions. The significantly increased numbers of nodules and short primary roots of *plenty* were rescued by the expression of *PLENTY* CDS in all three independent T_3_ transgenic lines, but not by the empty vector control (Fig. 1). The rescued primary root length was also observed in the non-inoculated condition (Fig. 1E, G). There were no significant differences in the number of first-order lateral roots (i.e. lateral roots emerging from the primary root) in the complemented plants (Supplementary Fig. S3). These results indicate that *PLENTY* functions not only in inhibiting nodulation and but also in primary root elongation, in both the presence and absence of rhizobia. As previously reported, *plenty* tends to form increased numbers of larger nodules (Yoshida *et al.*, 2010); therefore, we separately counted small nodules (<0.5 mm in diameter) and large nodules (>0.5 mm in diameter) to evaluate the complementation. The number of large nodules rather than small nodules was significantly reduced in the complemented lines (Fig. 1F).

### PLENTY is localized to the Golgi network

**Fig. 2.**
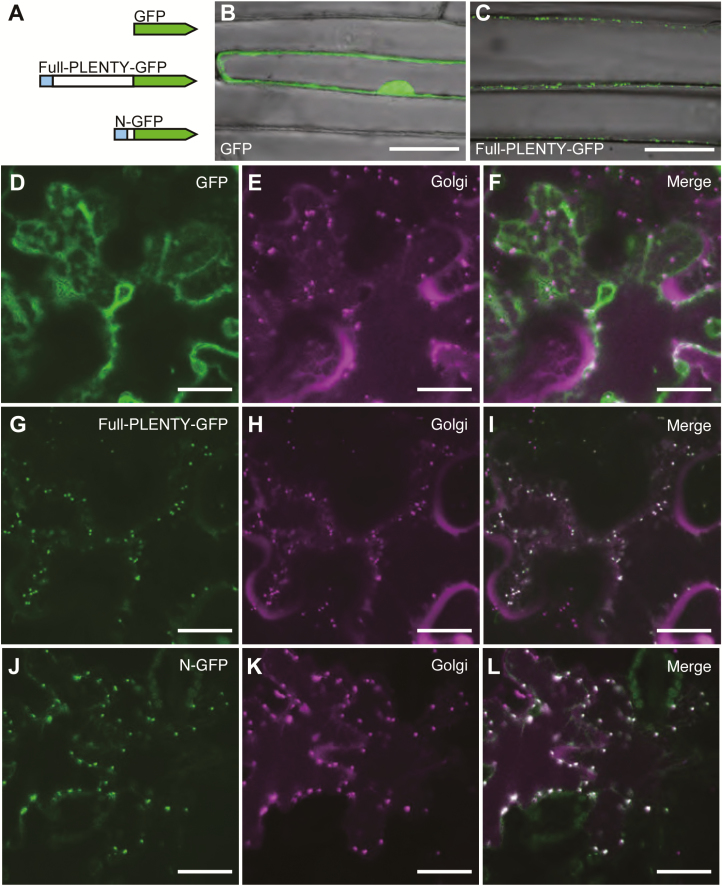
Subcellular localization of PLENTY–GFP fusion proteins. (A) Overview of the three GFP fusion protein constructs; GFP, Full-PLENTY–GFP, and N–GFP containing the first 58 amino acids of the N-terminal region of PLENTY. PLENTY has a putative secretory signal peptide at the N-terminus (shown in blue). (B–L) Confocal microscopic images of the localization of a series of PLENTY–GFP fusion proteins driven by the CaMV 35S promoter. (B, C) The transient expression in onion epidermal cells transformed using particle bombardment. (D–L) Transient expression in *N. benthamiana* pavement cells co-expressing the mCherry-fused *cis*-Golgi marker, transformed using *A. tumefaciens* infiltration. The constructs used for each analysis are shown in each panel. Merged images show the cytoplasmic localization of GFP (F) and the Golgi localization of Full-PLENTY–GFP and N–GFP (I, L). Scale bars=50 μm in (B, C) and 25 μm in (D–L). Similar GFP localization was observed in >10 transformed cells.

To assess the function of PLENTY, we next investigated its subcellular localization using a series of constructs expressing PLENTY–GFP fusion proteins. Since the N-terminal amino acid sequence of *Mt*RDN1 was previously predicted to be a signal peptide (SP) for the secretory pathway (Schnabel *et al.*, 2011), we generated three kinds of constructs fused to GFP: full-length PLENTY (Full-PLENTY–GFP), PLENTY lacking the first 46 amino acids of the N-terminal region including secretory peptides and the transmembrane domain (ΔN1-PLENTY–GFP), and only the first 58 amino acids of the N-terminal region of PLENTY (N–GFP) (Supplementary Appendix S2). We first examined the localization of GFP and Full-PLENTY–GFP constructs following their introduction into onion epidermal cells (Fig. 2B, C) and *L. japonicu*s root cells (Supplementary Fig. S4) using particle bombardment. Full-PLENTY–GFP was visible in the intracellular punctate structures. We next examined the *Agrobacterium* infiltration of *N. benthamiana* leaves and observed the same punctate localization of Full-PLENTY–GFP and N–GFP. To analyze these localization patterns in detail, we compared them with the localization of a mCherry-fused *cis*-Golgi marker, soybean α-1,2-mannosidase I (Nebenführ *et al*., 1999; Saint-Jore-Dupas *et al.*, 2006; Nelson *et al.*, 2007). The localization of Full-PLENTY–GFP and N–GFP overlapped with that of the *cis*-Golgi marker (Fig. 2D–L), as well as the previous localizations reported for its orthologs *At*HPAT1 and *Mt*RDN1 (Ogawa-Ohnishi *et al.*, 2013; Kassaw *et al.*, 2017). In addition, the Golgi localization of N–GFP, which contrasts with the cytoplasmic or nuclear localization of ΔN1-PLENTY–GFP (Supplementary Fig. S4), suggests that the N-terminal (SP) region is necessary and sufficient for targeting PLENTY to the Golgi.

### *In vitro* detection of PLENTY enzymatic activities

**Fig. 3.**
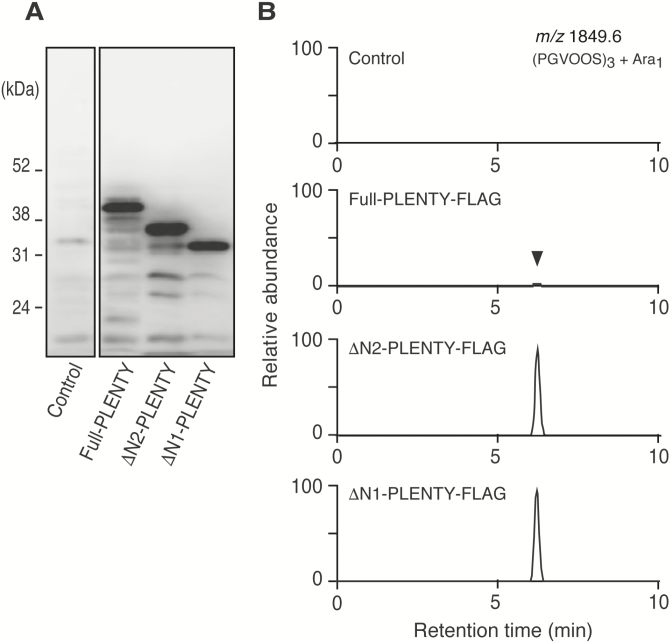
Identification of the HPAT activity of PLENTY *in vitro.* (A) Western blots of microsomal proteins in yeast expressing C-terminally FLAG-fused PLENTY proteins [Full-PLENTY (41.6 kDa), ΔN2-PLENTY with the first 25 amino acids deleted (38.8 kDa), or ΔN1-PLENTY with the first 46 amino acids deleted (36.4 kDa)], probed using an anti-FLAG antibody. (B) Identification of HPAT activity of the three recombinant PLENTY proteins. The synthetic substrate peptide (PGVOOS)_3_ was incubated with the FLAG-tag-fused recombinant proteins in the presence of UDP-β-l-Ara*f* and analyzed using LC/MS. The 1849.6 increase in *m/z* corresponds to the arabinosylation.

To determine whether PLENTY possess HPAT activity, we examined the enzymatic activity *in vitro* using yeast-expressed recombinant protein. A secretory signal and a transmembrane domain are predicted in the N-terminal region of PLENTY (Supplementary Appendix S2). Considering the difficulty in membrane protein purification, we designed not only Full-PLENTY but also two N-terminally deleted forms of PLENTY, ΔN1-PLENTY lacking the first 46 amino acids and ΔN2-PLENTY lacking the first 25 amino acids (Supplementary Appendix S2). Western blotting using an antibody to FLAG (anti-FLAG) demonstrated that all three recombinant proteins, Full-PLENTY (41.6 kDa), ΔN2-PLENTY (38.8 kDa), and ΔN1-PLENTY (36.4 kDa), were successfully expressed and collected in the microsomal membrane fractions (Fig. 3A). These fractions were then incubated with a synthetic tandem repeat peptide (PGVOOS)_3_, a previously developed substrate for detecting *At*HPAT activity based on the native PSY1 peptide in Arabidopsis (Amano *et al.*, 2007; Ogawa-Ohnishi *et al.*, 2013), in the presence of UDP-β-l-arabinofuranose (Ara*f*). A subsequent LC/MS analysis revealed that these PLENTY proteins catalyzed the arabinosylation of the peptide substrate (Fig. 3B).

### Hypernodulation of the *plenty* mutant was suppressed by the constitutive expression of *CLE-RS1* and *CLE-RS2* but not *CLE-RS3*

**Fig. 4.**
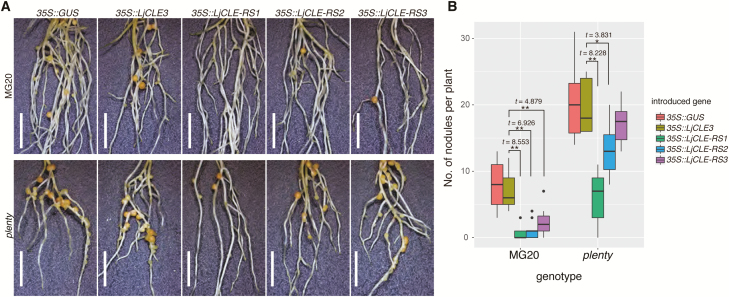
Hypernodulation of *plenty* was strongly suppressed by *CLE-RS1* and mildly suppressed by *CLE-RS2* but not by *CLE-RS3.* (A) Stereoscopic images of transgenic hairy roots constitutively expressing *GUS* and *LjCLE3* (as a control), *LjCLE-RS1*, *LjCLE-RS2*, or *LjCLE-RS3*. The constructs used for each analysis are shown in each panel. Scale bars=5 mm. (B) Boxplots of the number of nodules per individual transformed plant at 14 DAI with *M. loti* MAFF303099. The genotypes and introduced constructs are indicated on the graph. Statistical analyses were conducted using a two-tailed Welch’s *t*-test (**P<0.01, *P<0.05, *n*≥10). The black dots represent outliers. Experiments were performed in triplicate (*n*≥10 in each trial).

Although its native substrates have still not been identified, the HPAT activity of PLENTY led us to speculate that PLENTY modifies the CLE-RS1/2/3 peptides, because of their function in AON (Okamoto *et al.*, 2009; Nishida *et al.*, 2016). Hence, we investigated the nodule suppression effects introduced by the constitutive expression of *CLE-RS1/2/3* in the *plenty* mutant background using hairy root transformation. We hypothesized that if PLENTY mediates the arabinosylation of CLE-RS1/2/3, and if this modification is critical for their activity, the nodule suppression effect arising from the constitutive expression of *CLE-RS1/2/3* would be abolished in the *plenty* mutant, as was observed in *har1* (Okamoto *et al.*, 2009) and similarly for *CLE12* expression in *Mtrdn1* (Kassaw *et al.*, 2017). All three *35S::CLE-RS* (*35S::CLE-RS1/2/3*) constructs suppressed nodulation in the wild type in comparison with the negative controls *35S::GUS* and *35S::CLE3*. Unexpectedly, however, in *plenty*, the increased nodulation was significantly suppressed by two of the *CLE-RS* constructs, *35S::CLE-RS1* and *35S::CLE-RS2* (Fig. 4), although the degree of suppression was stronger in *35S::CLE-RS1* than in *35S::CLE-RS2*. Thus, the constitutive expression of *CLE-RS1/2* maintains sufficient biological activity to repress nodulation, even in *plenty*. On the other hand, the suppression effect of *35S::CLE-RS3* is completely abolished in *plenty*, indicating the involvement of PLENTY in the arabinosylation of CLE-RS3 rather than that of CLE-RS1/2.

### Increased nodulation is an additive phenotype in the *plenty har1* double mutant, compared with the single mutants

**Fig. 5.**
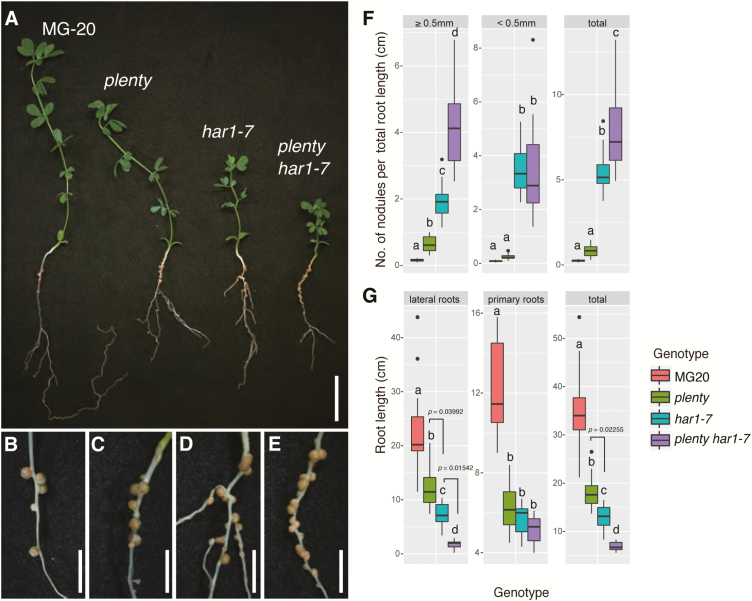
Additive nodulation of the *plenty har1-7* double mutant. (A) Nodulation in the wild-type (MG-20), *plenty*, *har1-7*, and *plenty har1-7* double mutant plants. (B–E) Magnified images of nodulated roots of the wild type (MG-20) (B), *plenty* (C), *har1-7* (D), and the *plenty har1-7* double mutant (E). (F) Boxplot of the nodule number [≥0.5 mm diameter (left), <0.5 mm diameter (middle), total (right)], normalized by the total root length of each plant, counted at 21 DAI with *M. loti* MAFF303099. (G) Boxplot of the lengths of lateral loots (left), primary roots (middle), and total roots (right) of each plant, measured for normalization in (F). Scale bars=2 cm in (A) and 5 mm in (B–E). The values of the total nodule numbers were used for the statistical analysis in (F). Different lower case letters represent statistically significant differences (*P*<0.05; Tukey’s HSD; *n*=14). 0.01<*P*<0.05 are denoted on the graph. The black dots represent outliers. Experiments were performed in triplicate (*n*≥10 in each trial).

Although is it unclear whether the CLE-RS1/2 peptides are substrates of PLENTY, the ligand–receptor interaction between CLE-RS1/2 and HAR1 has been clearly defined previously (Okamoto *et al.*, 2009, 2013; Sasaki *et al.*, 2014), as the inhibition of nodulation introduced by the constitutive expression of *CLE-RS1/2* occurs in a HAR1-dependent manner. To investigate the genetic interaction between PLENTY and HAR1 further, we generated a *plenty har1-7* double mutant and determined the nodule numbers normalized to the total root length of each plant (Fig. 5). The *har1-7* mutation resulted in the loss of both the transmembrane and kinase domains of HAR1; thus, *har1-7* is a possible null mutant (Magori *et al.*, 2009). The number of large nodules >0.5 mm in diameter and the total number of nodules in *plenty har1-7* were significantly increased relative to those of the *plenty* or *har1-7* single mutants (Fig. 5F). This indicates that PLENTY and HAR1 function in at least partially separate AON pathways. Notably, while the *plenty*, *har1-7*, and *plenty har1-7* mutants all had similar primary root lengths, an additive effect was observed for the shortening of the lateral roots in *plenty har1-7* (Fig. 5G), indicating their involvement in genetically non-overlapping pathways for lateral root elongation. Additionally, the number of first-order lateral roots in the inoculated condition was increased in *har1-7*, as previously reported (Szczyglowski *et al.*, 1998; Wopereis *et al.*, 2000), but decreased in *plenty har1-7*, suggesting that the *plenty* mutation suppressed not only lateral root elongation but also lateral root emergence in *har1-7* (Supplementary Fig. S3).

## Discussion

In this study, we cloned *LjPLENTY*, an ortholog of *MtRDN1* and *PsNOD3*, and a homolog of the three *AtHPAT* genes, and found that, like its orthologs, *Lj*PLENTY localizes to the Golgi complex (Ogawa-Ohnishi *et al.*, 2013; Kassaw *et al.*, 2017). Hydroxyproline *O*-arabinosylation is widely observed in secreted Arabidopsis peptides (Shinohara and Matsubayashi, 2010; Matsubayashi, 2014; Kucukoglu and Nilsson, 2015), and studies of HPAT homologs in Arabidopsis, tomato (*Solanum lycopersicum*), and the moss *Physcomitrella patens* have shown that the substrates of these enzymes are involved in diverse aspects of plant development, such as cellular tip growth and meristem maintenance (Ogawa-Ohnishi *et al.*, 2013; Xu *et al.*, 2015; MacAlister *et al.*, 2016). Additionally, the specific gene loss of the group 1 HPATs in the Brassicaceae (Supplementary Fig. S2) (Schnabel *et al.*, 2011) may be associated with the loss of arbuscular mycorrhizal symbiosis in these species (Delaux *et al.*, 2014).

Prior to the discovery of its molecular entity, *Ps*NOD3 was hypothesized to be involved in generating an unknown root-derived systemic signal that inhibited nodulation based on the findings of a series of grafting experiments (Postma *et al.*, 1988; Caetano-Anollés and Gresshoff, 1990; Li *et al.*, 2009; Novák, 2010). In particular, the hypernodulation on the adventitious roots originating from the wild-type scions on *nod3* rootstocks indicated that the decreased production of the systemic signal affected both wild-type and *nod3* roots. These previous reports also support the subsequent identification of CLE peptides as the root-derived signal. The nodule suppression by the constitutive expression of *MtCLE13* was found to be dependent on *Ps*NOD3 (Osipova *et al.*, 2012), and a separate study showed that the nodule suppression by *35S::MtCLE12*, but not *35S::MtCLE13*, was dependent on *MtRDN1* (Kassaw *et al.*, 2017). These findings suggest the hypothesis that *Lj*PLENTY participates in the maturation of at least one of the *Lj*CLE-RSs. In this study, stronger, milder, and no repression of nodulation was found by *35S::CLE-RS1*, *35S::CLE-RS2*, and *35S::CLE-RS3*, respectively, in the *plenty* mutant background. Based on the differential nodule suppression levels, we can only state the order of likelihood that each CLE-RS is the substrate of PLENTY: CLE-RS3 >CLE-RS2 >CLE-RS1 (Supplementary Fig. S5).

The differential requirements for PLENTY between the *CLE-RS* genes are similar to the differential requirements for RDN1 in the functions of *35S::MtCLE12/13* in *Medicago* (Kassaw *et al.*, 2017). The orthologous relationships of the CLEs are not clear because of their short amino acid sequences (Hastwell *et al.*, 2015, 2017), and because the core sequences in the CLE domains of *Lj*CLE-RS1/2 are shared more with *Mt*CLE13 than with *Mt*CLE12 (Imin *et al.*, 2018). This means it is difficult to determine whether the differences in their enzyme–substrate specificities are caused by differences in their amino acid sequences. *Mt*CLE13 did not suppress nodulation in *nod3* plants, suggesting the requirement for *Ps*NOD3 (Osipova *et al.*, 2012); but further studies using *Ps*CLEs rather than *Mt*CLE13 are needed for an understanding of the substrate–enzyme specificity in pea. Despite the successful detection of the enzymatic activities of PLENTY using artificially synthesized peptides, it is still unknown whether the arabinosylation of CLE-RS1/2/3 was performed by PLENTY, because the relevant results were based solely on the constitutive expression analysis. So far, the arabinosylation has been detected successfully only in CLE-RS2 (Okamoto *et al.*, 2013); therefore, whether CLE-RS1/3 are arabinosylated has also remained obscure (Supplementary Fig. S5). In conclusion, we cannot completely exclude the possibility that *Lj*CLE-RS1/2 are the substrates of PLENTY, but the CLE-RS1/2/3-HAR1 signaling pathway can be divided into PLENTY-dependent and PLENTY-independent pathways, namely PLENTY-independent for CLE-RS1, partially dependent for CLE-RS2, and strongly dependent for CLE-RS3 peptides (Supplementary Fig. S5). To evaluate the contribution of various enzymes to the modification of the CLE peptides accurately, assays to detect the modification levels of *in vivo* native peptides in respective single, double, and triple mutants of the three PLENTY paralogs should be performed in future studies. Alternatively, a loss-of-function analysis of the *CLE-RS* genes together with the *PLENTY* paralogs will provide important information.

Based on the additive nodules of the *plenty har1* double mutant, we propose that PLENTY and HAR1 at least partially function in separate AON pathways. First, this additive phenotype would be caused by the milder hypernodulation of *plenty* rather than that of *har1*. This milder phenotype may be affected by the functional redundancy among the three paralogs, as previously suggested for other species (Ogawa-Ohnishi *et al.*, 2013; MacAlister *et al.*, 2016; Kassaw *et al.*, 2017). Thus, the PLENTY-independent nodule suppression by the constitutively expressed *CLE-RS1/2* may be dependent on PLENTY2/3. As we expected, the expression patterns of *PLENTY2* and *PLENTY3* during nodulation were similar to that of *PLENTY* (Supplementary Figs S2, S6), as was previously shown for *MtRDN2/3* (Schnabel *et al.*, 2011). Nevertheless, the increased number of nodules in the *plenty har1-7* double mutant raises the possibility that PLENTY provokes AON independently of HAR1. In fact, the *Mt*SUNN-independent AON pathway has been discussed before (Kassaw *et al.*, 2015), based on the persistent suppression of excessive nodule formation in *Mtsunn*; however, alternative receptors functioning in a completely HAR1-independent manner have not yet been identified. All known candidate receptors for CLE peptides, *Lj*KLV, *Lj*CLV2/*Mt*CLV2/*Ps*SYM28, and *Lj*CRN/*Mt*CRN, have consistently been thought to interact with *Lj*HAR1/*Mt*SUNN/*Ps*SYM29 in the same genetic pathway (Miyazawa *et al.*, 2010; Krusell *et al.*, 2011; Crook *et al.*, 2016). We therefore postulate that other unknown LRR-RLKs function in the PLENTY-dependent and HAR1-independent pathway (Supplementary Fig. S5).

Finally, we considered the potential substrates of PLENTY functioning in a HAR1-independent manner. Aside from CLE-RS1/2/3, the most plausible candidates are the other CLE peptides, including *Lj*CLE40 (Nishida *et al.*, 2016; Hastwell *et al.*, 2017), the C-TERMINALLY ENCODED PEPTIDES (CEPs), and other related peptides found to be involved in nodulation in *Medicago* (de Bang *et al.*, 2017; Patel *et al.*, 2017); in particular, *Mt*CEP1 functioning in promoting nodulation under nitrogen-limited conditions (Imin *et al.*, 2013; Mohd-Radzman *et al.*, 2016). Moreover, the tri-arabinosylation of the *Mt*CEP1 proline reduced or eliminated the nodule-promoting effect of this peptide (Patel *et al.*, 2017), which contrasts with the necessity of arabinosylation for *Lj*CLE-RS2 or *Mt*CLE12/13 activities (Okamoto *et al.*, 2013; Imin *et al.*, 2018). *Mt*RDN1 may therefore keep *Mt*CEP1 inactive to inhibit increased nodulation; thus, the *Mt*CEP1 ortholog may actually be a substrate of PLENTY. Additionally, the inhibition of lateral root emergence by *Mt*CEP was abolished by non-arabinosylated *Mt*CEP (Patel *et al.*, 2017). The reduced number of emerged lateral roots of the *plenty har1-7* may be caused by accumulation of the non-arabinosylated CEP1-like peptide (Supplementary Fig. S3). Also, other unidentified substrates of PLENTY may have effects on the shorter primary root of *plenty* (Fig. 1) or the shorter and reduced lateral root of *plenty har1-7* (Fig. 5; Supplementary Fig. S3). The arabinosylated peptides involved in root architecture are strong candidates to be these substrates (Corcilius *et al.*, 2017; Patel *et al.*, 2017; Oh *et al.*, 2018). Interestingly, the *Mt*CEP1 receptor, compact root architecture 2, acts on the shoot for nodulation but on the root for lateral root development through different pathways (Huault *et al.*, 2014; Mohd-Radzman *et al.*, 2016). *Lj*HAR1 is also a common factor involved in both nodulation control and non-symbiotic root development (Wopereis *et al.*, 2000), but its ligand and shoot/root dependency responding to symbiotic and non-symbiotic phenotype were unknown. Identifying substrates of PLENTY will lead to a further understanding of how nodulation and root architecture are controlled at the same time via HAR1 or other receptors.

## Supplementary data

Supplementary data are available at *JXB* online.

**Fig. S1.** Identification of the *plenty* locus.

**Fig. S2.** Phylogenetic tree of the *PLENTY* family in land plants.

**Fig. S3.** First-order lateral roots in the complementation test and the *plenty har1-7* double mutant analysis.

**Fig. S4.** The N-terminal region of PLENTY is necessary and sufficient for localization to the Golgi.

**Fig. S5.** A working model of PLENTY in the negative control of nodulation.

**Fig. S6.** The gene expression patterns of the *PLENTY* paralogs.

**Table S1.** Newly developed genetic markers for the map-based cloning of *PLENTY.*

**Table S2.** Primers used in this study.

**Appendix S1.** FASTA file of amino acid sequences used for the phylogenetic analysis.

**Appendix S2.** The deletion series of PLENTY proteins used in this study.

## Supplementary Material

Supplementary Tables

Supplementary Figures

Supplementary Appendix
