# Supplementary material for: PLENTY, a hydroxyproline O-arabinosyltransferase, negatively regulates root nodule symbiosis in Lotus japonicus
Source: J Exp Bot. 2018 Oct 23;70(2):507–17. doi: 10.1093/jxb/ery364 (PMC6322572; doi:10.1093/jxb/ery364)
Supplement: Supplementary Tables [file ery364_suppl_supplementary_tables.pdf]

Table S1. Newly developed genetic markers for the map-based cloning of *PLENTY*

Marker	Clone	Sequence before polymorphisms	Polymorphism		Sequence after polymorphism	Primers for detecting polymorphism		Restriction enzyme	Length of fragment (bp)
			MG-20	B-129		Direction	Sequence		
EY004	LjT42O06	GCGGGTAAGGCAACAACTCA	CCTGAGACTAATTCCTCATC	-	ACACGCAATCTTACTTTGAA	F	TGGTGAGGTGGAAGTGTCAA	-	MG-20; 184
						R	CCAATGCCTGCTAGTTTGCT		B-129; 164
EY005	LjT42O06	AAACACACATACATCCCCTT	A	G	CACCTAAATTGTTCTCTAAG	F	CAAACACACATACATCCC**GA**TC	PvuI	MG-20; 170
						R	GCAGAAATGAATTTGAATGG		B-129; 149+21

Table S2. Primers used in this study

Use	Primer name	Sequence	Notes	Reference
Real-time RT-PCR	EF1a_F	GCAGGTCTTTGTGTCAAGTCTT		Groth *et al.*, 2010
	EF1a_R	CGATCCAGAACCCAGTTCT		Groth *et al.*, 2010
	PLENTY_5UTR_F	GACTTTGCCCTTCCCATGTTTTT		
	PLENTY_5UTR_R	TTAGTTCTAAGCAATTGCTGATAGCAGA		
	PLENTY2_5UTR_F	AAGTAATGGTGAAGCTCGCGC		
	PLENTY2_5UTR_R	TCTTCTGCTGAAGTTGTGGGAAAATT		
	PLENTY3_5UTR_F	TTGCCACTACAACCCATGTTGATT		
	PLENTY3_5UTR_R	ATGTAAGGAACACCCCTCCAAC		

	CLE-RS1_F	TGCAAGTGTCGATGCTCATAGC		Okamoto *et al.*, 2009
	CLE-RS1_R	GATGTTTTGCTGAACCAAGGGATA		Okamoto *et al.*, 2009
	CLE-RS2_F	GCTCGTAATCTCCAAATCATTCACA		Okamoto *et al.*, 2009
	CLE-RS2_R	GGTGAGAGTCTTTGCTGTTGATATCC		Okamoto *et al.*, 2009
Construction	PLENTY_CDS_F	ATGGGAAGGGCAAAGTCACTTC	Cloning into the pGEM®-T-Easy vector	
	PLENTY_CDS_R	TCAGCTTCTATTTAATGAATCCCATTCAG	Cloning into the pGEM®-T-Easy vector	
	N_PLENTY_CDS_R	ATGGTATTTCGCATTAGTGCTTG	Cloning into the pGEM®-T-Easy vector	
	□N1_PLENTY_CDS_F	ATGTTGGGTTCGGCAAGCAC	Cloning into the pGEM®-T-Easy vector	
	mut_vector_F	GCGGCCGCCTGCAGGTCG	Introduce mutations for GFP-fusion	
	mut_PLENTY_tail_R	GCTTCTATTTAATGAATCCCATTCAGG	Introduce mutations at the tail of PLENTY and □N-PLENTY for GFP-fusion	
	mut_N-PLENTY_tail_R	GTGATTATGGTATTTCGCATTAGTGC	Introduce mutations at the tail of N-PLENTY for GFP-fusion	
	full_PLENTY_inf_F	TACCGAGCTCGGATCATGGGAAGGGCAAAGTCA	Cloning into pYES2 for yeast expression system	
	□N2_PLENTY_inf_F	TACCGAGCTCGGATCATGATCATGGACCATAGG	Cloning into pYES3 for yeast expression system	
	□N1_PLENTY_inf_F	TACCGAGCTCGGATCATGTTGGGTTCGGCAAG	Cloning into pYES4 for yeast expression system	
	PLENTY_FLAG_inf_R1	TTACTTGTCGTCATCGTCCTTGTAGTCGCTTCTATTTAATGAATC	Cloning into pYES5 for yeast expression system	
	PLENTY_FLAG_inf_R2	CGTTACTAGTGGATCTTACTTGTCGTCATCGTCCTTGTAGTC	Cloning into pYES6 for yeast expression system	
Genotyping	plenty_F	ATGGGAAGGGCAAAGTCACTTC		
	plenty_R	CCATCAACTGGTCTGTCCTT		
	HPTII_F	GGCGAGTACTTCTACACAGC	For genotyping stably transformed plants	
	HPTII_R	CATGTGTATCACTGGCAAAC	For genotyping stably transformed plants	
Deleted region	PLENTY_del_down_F	CATAAGGCATCCCTGC		

detection				
	PLENTY_del_down_R1	AAGACTCCTGGACTGCAAGA		
	PLENTY_del_down_R2	AAGATATGTGCCTTGTACGAGTG		
	PLENTY_del_down_R3	ATCCATGCCGAAATTCAGC		
	PLENTY_del_down_R4	AAGGATCCTGCAGCTGCAA		
	PLENTY_del_up_F1	TTCACAGGTGTAGCCCTGT		
	PLENTY_del_up_F2	TAACATCCTAATAATGAAATTTATAACTTATCAATC		
	PLENTY_del_up_F3	CACAGGCCAAGTTAGTGACG		
	PLENTY_del_up_F4	CGCTCCGAGATTAATTCTCACTTA		
	PLENTY_del_up_R	TTTGAGGTTTGCCATGGTTGAGAT		
5' or 3' RACE	3RACE_PLENTY_1st	GTAAATCCTTTGCCTAATTTGGCT		
	3RACE_PLENTY_2nd	CTAGAACCCAACCAGCAGG		
	5RACE_PLENTY_2nd	CAGTAACGGGACCCTTGTC		
	5RACE_PLENTY_1st	GGAGAATTGCCAATCGGATC		
	3RACE_PLENTY2_1st	GCCTGATCACGTATTTGTACG		
	3RACE_PLENTY2_2nd	TCCCAATCTGGCTTATGGAG		
	5RACE_PLENTY2_2nd	TGTTACTGGTCCTTTCTCCTC		
	5RACE_PLENTY2_1st	GAGAATTGCCAATCGGATCAATA		
	3RACE_PLENTY3_1st	GCCCGATCATATAATTGTCAAAC		
	3RACE_PLENTY3_2nd	TACCCAACTTAGCTAAAGATGGG		
	5RACE_PLENTY3_2nd	CCATTGGGACACTCCGTAA		
	5RACE_PLENTY3_1st	CCAACAATAACTGGTGAATTTCC		
References				
**Groth, M., *et al.*** (2010) *NENA*, a *Lotus japonicus* homolog of Sec13, by arbuscular mycorrhiza fungi and rhizobia but dispensable for cortical endosymbiotic development. *Plant Cell*, 22, 2509-2526.				
**Okamoto, S., *et al.*** (2009) Nod Factor/Nitrate-Induced CLE Genes that Drive HAR1-Mediated Systemic Regulation of Nodulation. *Plant and Cell Physiology*, 50, 67-77.				
