# Supplementary material for: PLENTY, a hydroxyproline O-arabinosyltransferase, negatively regulates root nodule symbiosis in Lotus japonicus
Source: J Exp Bot. 2018 Oct 23;70(2):507–17. doi: 10.1093/jxb/ery364 (PMC6322572; doi:10.1093/jxb/ery364)
Supplement: Supplementary Figures [file ery364_suppl_supplementary_figures.pdf]

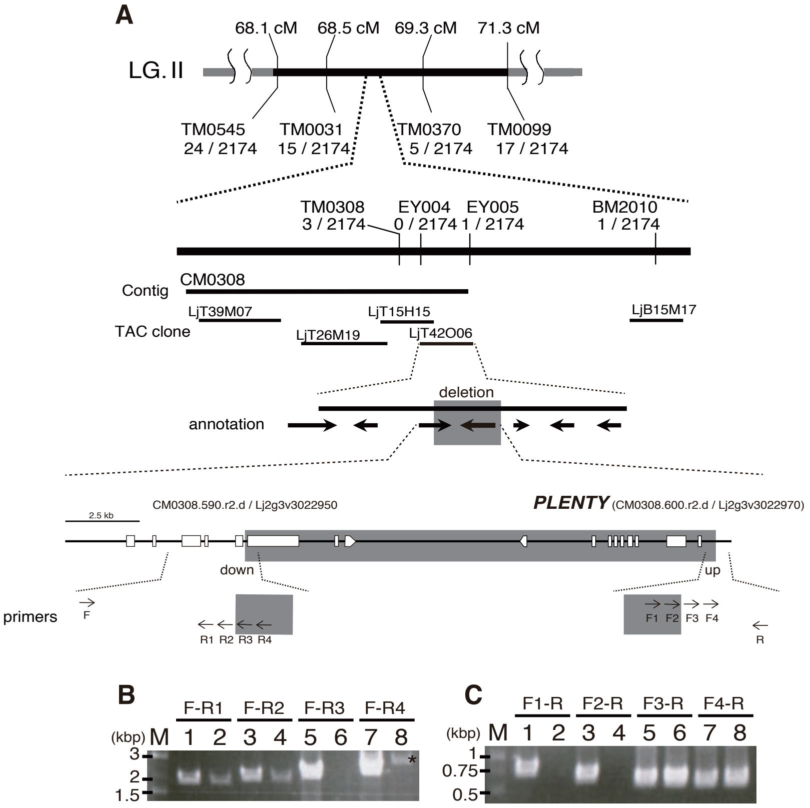

Figure S1. Identification of the *plenty* locus.

(A) The locus containing *PLENTY* on linkage group II is shown, along with the number of recombination events (events/total chromosomes) and the physical map with names of the TAC/BAC clones. The large deletion (shaded in grey), which covered the genes CM0308.590.r2.d/Lj2g3v3022950 and CM0308.600.r2.d/Lj2g3v3022970 (*PLENTY*), was detected using genomic PCR in the chromosomal region between TM0308 and EY005 (a newly developed marker for this study; see Table S1). Arrows indicate the primers used to identify the approximately 16-kb deletion (Table S2). (B, C) Agarose gel electrophoresis of DNA markers (M) and the PCR products of genomic DNA templates of the wild-type line MG-20 (lanes 1, 3, 5, 7) and *plenty* (lanes 2, 4, 6, 8). The DNA fragments were amplified using sets of primers as shown in each lane, and their positions are shown in (A). The asterisk indicates non-specific amplification that we checked nucleotide sequence by sequencing analysis.

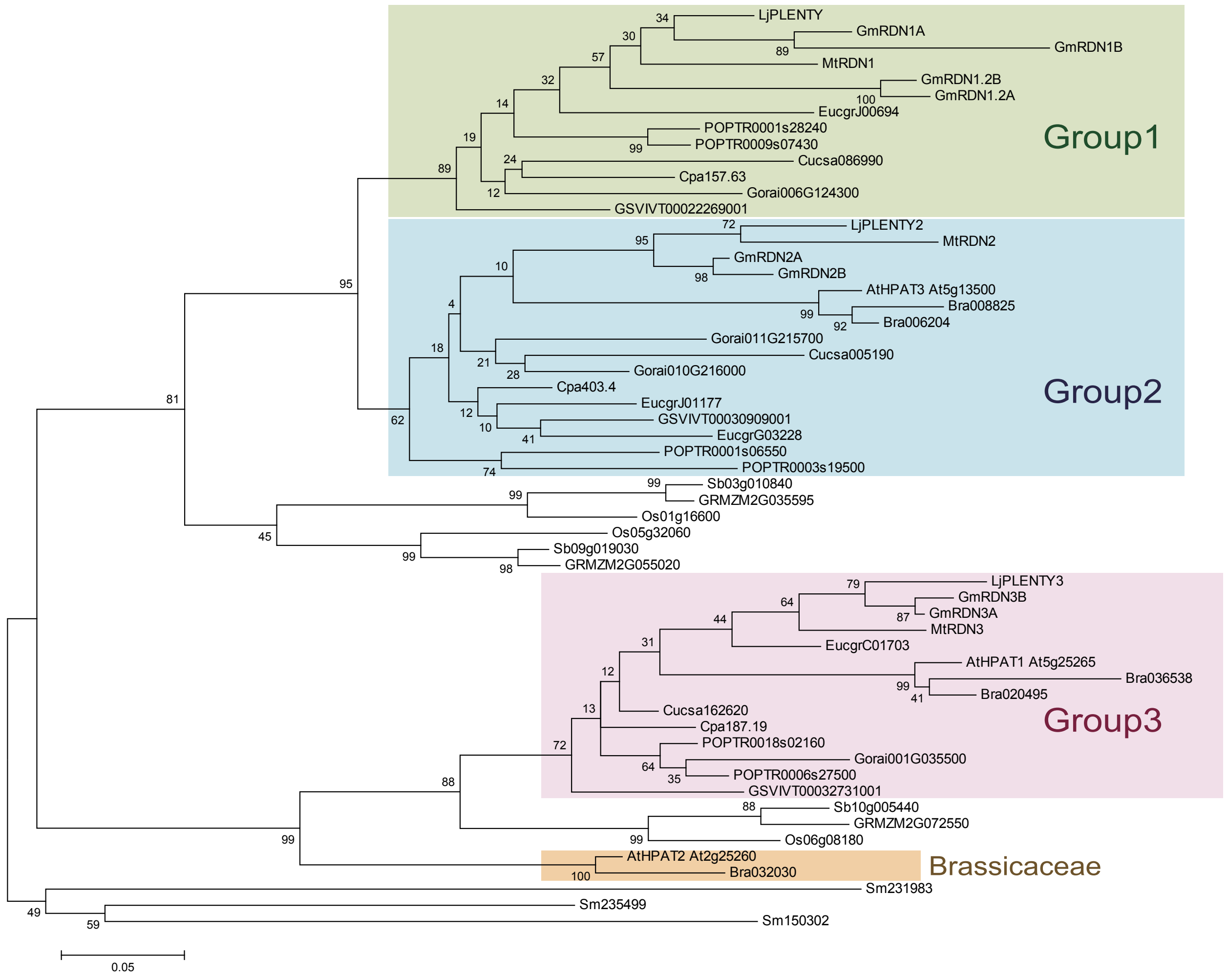

Figure S2. Phylogenetic tree of the *PLENTY* family in the land plants.

The tree is drawn to scale, with branch lengths measured in the number of substitutions per site (5% sequence divergence). The side bar displays 0.05 substitutes per amino acid position. The 57 amino acid sequences used for this analysis consist of the same data set used in the study of *Mt*RDN1 (Schnabel et al., 2011), including the eudicots *Lotus japonicus* (Lj), *Medicago truncatula* (Mt), soybean (*Glycine max*, Gm), grape (*Vitus vinifera*, GSVIVT), cucumber (*Cucumus sativus*, Cucsa), poplar (*Populus trichocarpa*, POPTR), and *Arabidopsis thaliana* (*At*); the monocots rice (*Oryza sativa*, Os), sorghum (*Sorghum bicolor*, Sb), and maize (*Zea mays*, GRMZM); and the lycophyte *Selaginella moellendorfii* (Sm). Sequences from Phytozome (http://www.phytozome.net/) were also added, including *Brassica rapa Chiifu-401* (Bra), papaya (*Carica papaya*, Cpa), cotton (*Gossypium raimondii*, Gorai), and eucalyptus (*Eucalyptus grandis*, Eucgr) (see Supplementary Appendix S1). All positions containing gaps and missing data were eliminated. A total of 266 positions comprised the final dataset. The percentage of trees in which the associated taxa are clustered together following bootstrapping (1,000 replicates) is shown next to the branches. Evolutionary analyses were conducted in MEGA6 (Tamura *et al.*, 2013). All *PLENTY* homologs from the eudicots, except for those from *Arabidopsis thaliana* and *Brassica rapa* (orange), were classified into three groups, Group 1 (green), Group 2 (blue), and Group 3 (pink).

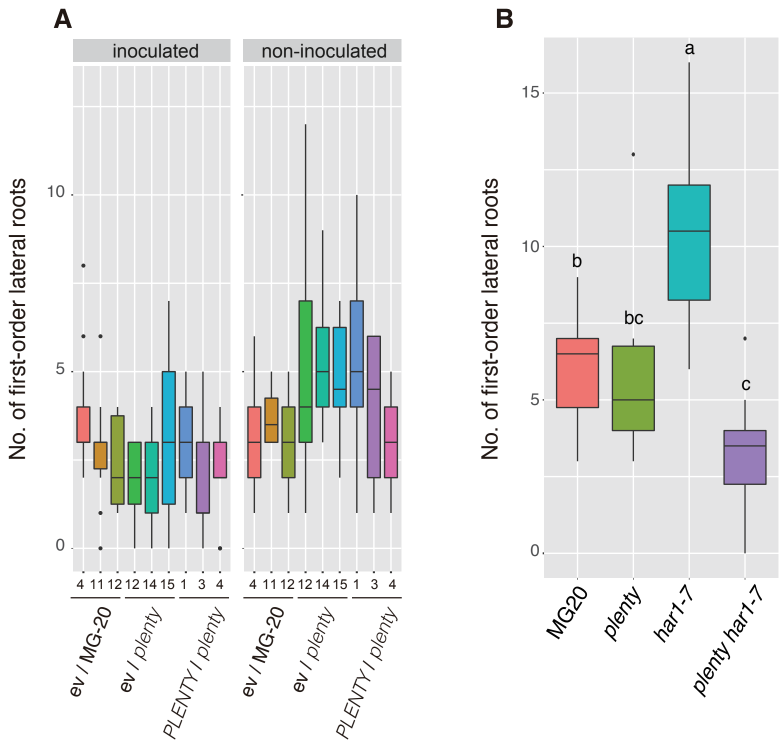

Figure. S3. First-order lateral roots in the complementation test and the *plenty har1-7* double mutant analysis.

(A) Boxplots of the number of first order lateral roots of T3 transgenic plants expressing the empty vector pUB-GW-GFP (ev) or pUB-GW-Full-PLENTY (*PLENTY*) at 14 DAI with *M. loti* MAFF303099 (left) or at 21 DAG without rhizobia (right). T3 transformants are the same as that in Figure 1 (n ≥ 10). (B) Boxplots of the number of first order lateral roots of MG-20 wild type, *plenty*, *har1-7*, and *plenty har1-7* at 21 DAI (n=14). Respective plants are the same as that in Figure 5. Different lowercase letters represent statistically significant differences (P<0.05; Tukey's HSD).

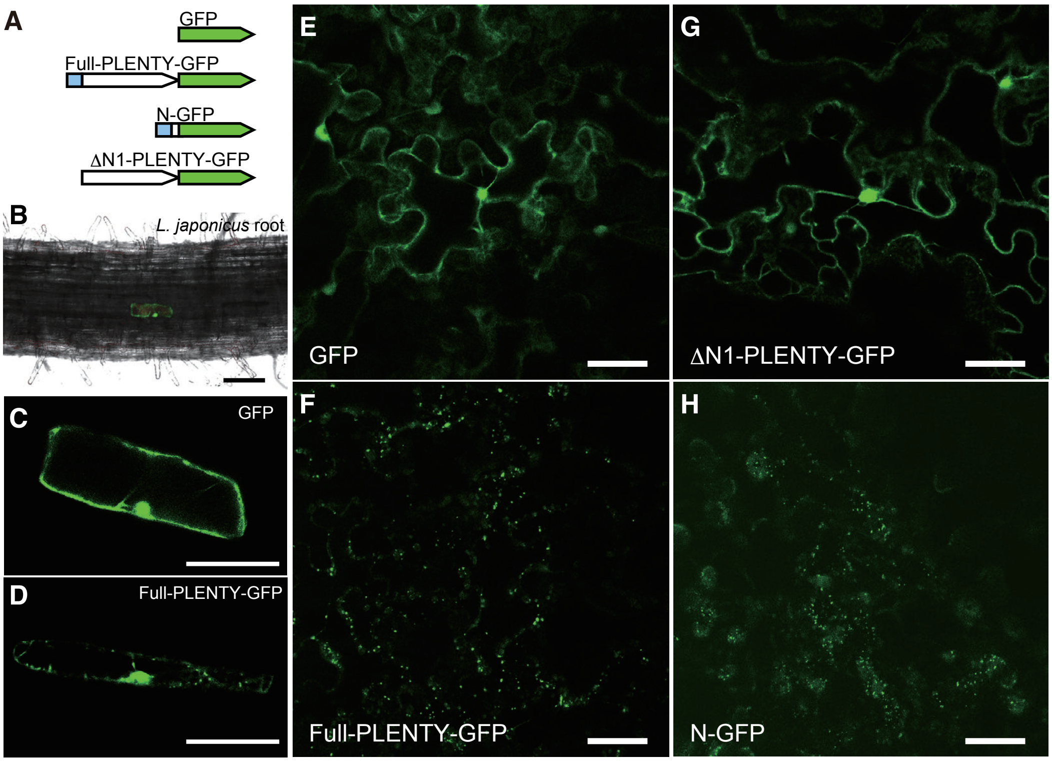

Figure S4. The N-terminal region of PLENTY is necessary and sufficient for localization to the Golgi.

(A) Overview of the four GFP-fusion protein constructs, Full-PLENTY-GFP, N-GFP (N-terminal domain only), ΔN1-PLENTY-GFP (lacking the N terminus), and the GFP control. PLENTY includes a putative secretory signal peptide (SP) at the N-terminus (SP; shown in blue). (B–D) Confocal microscopic GFP fluorescence images of the GFP-fusion proteins in *Lotus japonicus* root cells transformed by particle bombardment. (E–H) Confocal images of the GFP-fusion proteins in *N. benthamiana* pavement cells transformed by Agrobacterium infiltration. Constructs used for the analysis are shown in each panel. Scale bars = 100 μm in (B) and 50 μm in (C–H).

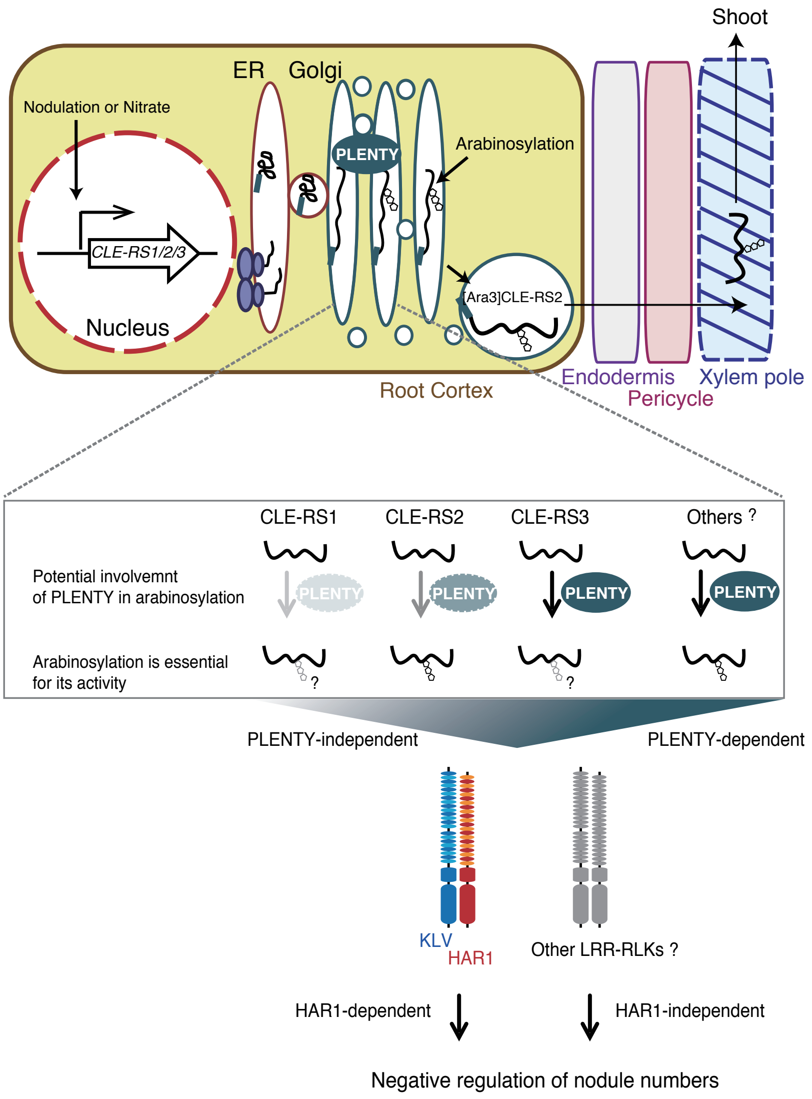

Figure S5. A working model of PLENTY in the negative control of nodulation.

The best plausible model for the generation of arabinosylated peptides (i.e., CLE-RS2) via PLENTY. *CLE-RS1/2/3* gene expression are induced by prior nodulation or nitrate and post-translationally modified by PLENTY. These modified peptides are secreted into xylem. This model is based on that previously proposed (Suzaki *et al.*, 2015). The differential requirements for PLENTY between the *CLE-RS*s are shown in a gradation. CLE-RS1/2/3-HAR1 signaling pathway can be divided into PLENTY-dependent and PLENTY-independent pathway. Moreover, other substrates for PLENTY functioned in HAR1-independent pathway are predicted based on *plenty har1* double mutant phenotype.

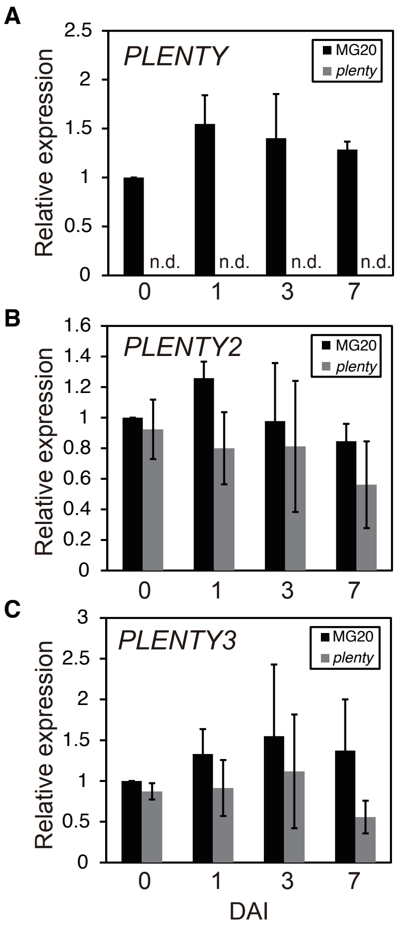

Figure S6. The gene expression patterns of the *PLENTY* paralogs.

RT-qPCR analysis of *PLENTY* (A), *PLENTY2* (B), and *PLENTY3* expression (C), normalized to the expression level of *EF-1a* in the wild type Miyakojima MG-20 and *plenty*, in non-inoculated plants (0), and at 1, 3, and 7 DAI with *M. loti* MAFF303099. Fold changes in expression are shown relative to that of the MG-20 wild type at 0 DAI. Data are the mean ± SD of three biological replicates. Data are the mean ± SD of three technical replicates. n.d. means not detected.
