# Supplementary material for: PLENTY, a hydroxyproline O-arabinosyltransferase, negatively regulates root nodule symbiosis in Lotus japonicus
Source: J Exp Bot. 2018 Oct 23;70(2):507–17. doi: 10.1093/jxb/ery364 (PMC6322572; doi:10.1093/jxb/ery364)
Supplement: Supplementary Appendix [file ery364_suppl_supplementary_appendix.pdf]

Appendix S1. FASTA file of amino acid sequences used for the phylogenetic analysis

>LjPLENTY

MIVRKNMGRAKSLLMLLMVLGFFFATYNLVSMIMDHRAGNWVADGLESFDRKMLGSASTNAKYHVALTATDAAYSQWQCRIMYYWYKKVKDMPGSNMGKFTRILHSGRTDQLMDEIPTFVVDPL

PEGLDRGYIVLNRPWAFVQWLEKADIEEEYILMAEPDHIFVNPLPNLASRTQPAGYPFFYIKPAENEKIIRKFYPKDKGPVTDVDPIGNSPVIIQKSLIEEIAPTWVNVSLRMKDDPETDKAFG

WVLEMYAYAVASALHGVKHILRKDFMLQPPWDRHVGKTFIIHYTYGCDYNLKGELTYGKIGEWRFDKRSYLMGPPPKNLSLPPPGVPESVVRLVKMVNEATANIPEWDSLNRS

>LjPLENTY2

MARASPLLIIFLVFGSSFATYNLVTMIIRYGSSESVATDDGALFFDPIIEMPDHVKNRKTSKAPFHVALTATDAPYNQWQCRIMYYWYKRQRNSPGSEMGGFTRILHSGKPDNLMDEIPTVVVD

PLPAGLDRGYIVLNRPWAFVQWLEKAKIEEEYVLMAEPDHVFVRPLPNLAYGENPAAFPFFYIRPDQNEKIIRKNYPEEKGPVTNIDPIGNSPVIIKTDVIAKIAPTWMNVSLKMKEDPETDKA

FGWVLEMYAYAIASALHGVRHILRKDFMLQPPWDLETHNKYIIHYTYGCDYNLKGELTYGKIGEWRFDKRSHLRGPPPRNLPLPPPGVPESVVTLVKMVNEATANIPHWDTP

>LjPLENTY3

MGCSGNLFFTILITFSVALITYNIIISGNAPLRQDFPGPSRRPTITIDPIIEMPLRRHSSSSKRLFHTAVTASDSVYNTWQCRVMYHWFKKFQADPDSSMGGFTRILHSGKPDAFMDEIPTFVA

QPLPSGMDQGYIVLNRPWAFVQWLQQADIKEDYILMSEPDHIIVKPIPNLAKDGMGAAFPFFYIEPKKYETVLRKYFPEENGPVTNIDPIGNSPVIVGKESLKKIAPTWMNVSLAMKKDPETDK

AFGWVLEMYAYAVASALHGVRNILYKDFMIQPPWDKEIGKSYIIHYTYGCDYNMKGELTYGKIGEWRFDKRSYDHVAPPKNLTLPPPGVPESVVTLVKMVNEATASIPNWYS

>MtRDN1

MIVRKSMGRVKSLLMLLMVLGFSFATYNLVFMMMEHKAGNDLGSFDGKAMEIRNTNSKYHVAVTATDAAYSQWQCRIMYYWYKKTKDMPGSAMGKFTRILHSGRGDQLMNEIPTFVVDPLPEGL

DRGYIVLNRPWAFVQWLEKAVIDEEYILMAEPDHIFVNPLPNLATENEPAGYPFFYIKPAENEKIMRKFYPKENGPVTDVDPIGNSPVIIHKYMLEEIAPTWVNISLRMKDDPETDKAFGWVLE

MYAYAVASALHGIKHILRKDFMLQPPWDLDVGKKFIIHFTYGCDYNLKGKTYGKIGEWRFDKRSYLMGPPPKNLSLPPPGVPESVVRLVKMVNEATANIPNWDSLNRS

>MtRDN2

MARASPLLMICLVLGSSFATYNLVTMIIHYGSADSLATEDGGLFFDPIVEMPEHVKNTKTSKAPFHIALTATDAIYNKWQCRIMYYWYKKQRSLPGSEMGGFTRILHSGKADNLMDEIPTVVVD

PLPEGLDRGYVVLNRPWAFVQWLEKANIEEEYILMAEPDHVFVRPLPNLAFGENPAAFPFFYIKPKENEKIVRKYYPEENGPVTNVDPIGNSPVIIRKDLIAKIAPTWMNISMKMKEDPETDKA

FGWVLEMYGYAVASALHGVRHILRKDFMLQPPWDTETFNKYIIHYTYGCDYNLKGELTYGKIGEWRFDKRSHLRGPPPRNLPLPPPGVPESVATLVKMVNEASANIPNWDTL

>MtRDN3

MGCGNMFFTILITFSVTLITYNIIISGNAPLKQDFPGPSRKPSIKIDPIIKMPLNRKSASSKRLFHTAVTASDSVYNTWQCRVMYYWFKKMKESGDENSGMGGFTRILHSGKSDQYMDEIPTFV

AQPLPSGMDQGYIVLNRPWAFVQWLQQADIKEDYILMSEPDHIIVKPIPNLARDGMGAAFPFFYIEPKKYEKVLRKYYPEENGPVTNIDPIGNSPVIVGKESLKKIAPTWMNVSLAMKKDPETD

KAFGWVLEMYAYAVSSALHGVGNILHKDFMIQPPWDKELGKTFIIHYTYGCDYSMKGELTYGKIGEWRFDKRSYDLVAP-KNLTLPPPGVPESVVTLVKMVNEAAANIPNWSS

>GmRDN1A

MIVRKGMGRAKSLLLLLMVLLFSFATYNLVAMIMDHKADGLESLNRKMMVSGKTNSKFHVAVTATDAAYNQWQCRIMYYWYKKVKDMPGSDMGKFTRILHSGRSDQLMDEIPTFVVDPLPEGLD

KGYIVLNRPWAFVQWLEKADIEEEYILMAEPDHI

FVKPLPNLAQGTLPAAFPFFYIKPDQNEKIIRKFYPEENGPVTDVDPIGNSPVIIKKSLMEEIASTWVNVSLRMKDDQETDKAFGWVLEMYAYAVASALHGVKHNLRKDFMLQPPWDLNVENKF

IIHYTYGCDYNMKGELTYGKIGEWRFDKRSYLLGPPPKNLPLPPPGVPESVVRLVKMVNEATANIPEWDSLNKS

>GmRDN1B

MVVRKSMGRAKSLLLLLMVLIFSFATYNLVAMIMNLKTDGSESFNRKIMRSGKTNSKFHVALTATDSPYSQWQCRIMYYWYKKVKDMPGSDMGKLTRILHSGRPDQLMDEIPTFVVHPLPRGLD

KGYVVLNRPWAFVQWLEKADIEEEYILMAEPDHIFVNPLPNLAHGTLPAAFPFFYMNSYENVDIIRKFYPEEKGPVTDVEPIGNSPVIIKKSLMEEIAPTWVNISLRMKNDPETDETFGWVLEM

YAYAVTSALHGVEHNLRKDFMLQPPWDENVENKFIIHYTYGCDYNMKGELTYGKIGEWRFDKRYHLLGPPPKNLPLPPPGVPETVVQLVKMVNEATANIPEWDSINNSRGKDDKP

>GmRDN1.2A

MIVRKNKPSARSLLMLFVMVLAFLFVTYNLVFIIKHHMGGTWVAEELRFLDPVISMPGKVMALANSNSKFHVAVTATDAAYSQWQCRIMYYWYKKVKDMPGSDMGKFTRIVHSGRQDQLMDEIP

TFVVDPLPEGLDRGYVVMNRPWAFVQWLEKADIEEEYILMAEPDHIFVNPLPNLTNGNQPAGYPFFYIKPVKHEKILRKFYPKANGPITAIDPIGNSPVIIQKSLLEEIAPTWVNISLQMKDDP

ATDETFGWVLEMYAYAVASALHGVRHILHDNFMLQPPWDFDVENNFIIHYTYACDYNLKGELTYGKIGEWRFNKRFYLSGPPPKNLSLPPPGVPESVVQLVKMINEATANIPKWDSLNRS

>GmRDN1.2B

MIVRKNKPSAKSLLVLFIMVLAFFFVTYNLVFMIKHHKGGSWVADELGFLDPLIRNIPGKVMVLANSNSKFHVAVTATDAAYSQWQCRIMYYWYKKVKDMPGSDMGKFTRIVHSGRPDQLMDEI

PTFVVDPLPAGLDRGYVVMNRPWAFVQWLEKADIEEEYILMAEPDHIFVNPXPNLTNGNQPAGYPFFYIKPVKHGKIIRKFYPKANGPITDIDPIGNSPVIIQKSLLEEIAPTWVNISLQMKDD

PETDETFGWVLEMYAYAVASALHGVRHILHDNFMLQPPWDFDVENKFIIHYTYACDYNLKGELTYGKIGEWRFNKRFYLTGPPPKNLSLPPPGVPESVVLLVKMINEATANIPKWDSLNRS

>GmRDN2A

MGRASSLLIIFLVLGSSFATYNVVTMIRHYGSSEGVAVNDGALFFDPITEMPDHVKNRKTSKVPFHVALTATDAPYNKWQCRVMYYWYKQQKKLPGSEMGGFTRILHSGNPDNLMDEIPTVVVD

PLPAGLDRGYIVLNRPWAFVQWLEKTKIEEEYVLMAEPDHIFVRPLPNLAYGGHPAAFPFFYIRPDENEKIIRKFYPEELGPVTNVDPIGNSPVIIRKDLIAKIAPTWMNISLKMKEDPETDKA

FGWVLEMYAYAVASALHGVRHILRKDFMLQPPWDLETNKKYIIHYTYGCDYNMKGELTYGKVGEWRFDKRSHLRGPPPKNLPLPPPGVPESVVTLVKMVNEASANIPNWDTS

>GmRDN2B

MGRASLLLIVFLVLGSSFATYNVVTMIRHYGSSEGVAVDDGALFFDPITEMPDHVKNRKTSKAPFHVALTATDAPYNKWQCRVMYYWYKQQKKLPGSEMGGFTRILHSGNPDNLMNEIPTVVVD

PLPAGLDRGYIVLNRPWAFVQWLEKTKIEEEYVLMAEPDHIFLRPLPNLAFGGHPAAFPFFYIRPDQNEKTIRKFYPEELGPVTNVDPIGNSPVIIRKDLIAKIAPTWMNISLKMKEDPETDKA

FGWVLEMYAYAVASAVHGVRHILRKDFMLQPPWDLETNKKYILHYTYGCDYNMKGELTYGKIGEWRFDKRSHLRGPPPKNLPLPPPGVPESVVTLVKMVNEASANIPNWDSSSTSS

>GmRDN3A

MGCGNLFFTVLITFSVALITYNIIISANAPLKQDFPGPSRPSIKVDPLIKMPLHRSSSSEKSKKRLFHTAVTASDSVYNTWQCRVMYYWFKKFRDGGGDESGMGGFTRILHSGKPDQFMDEIPT

FVAQPLPAGMDQGYIVLNRPWAFVQWLQQADIKEDYILMSEPDHIIVKPIPNLARDGLGAAFPFFYIEPKKYETVLRKYFPEEKGPITNIDPIGNSPVIVGKEFLKKIAPTWMNVSLAMKKDPE

TDKAFGWVLEMYAYAVASALHGVRNILYKDFMIQPPWDQEIGKTYIIHYTYGCDYTMKGELTYGKIGEWRFDKRSYDKVAPPKNLTLPPPGVPESVVTLVKMVNEATANIPNWWS

>GmRDN3B

MGCGNLFFTILITFSVALITYNIIISANAPLKQDFPGPSRPSIKVDPLIKMPLHRKSSSSEESKKRLLFHTAVTASDSVYNTWQCRVMYYWFKKVRDEGGDESGMGGFTRILHSGKPDQFMDEI

PTFVAQPLPAGMDQGYIVLNRPWAFVQWLQQADIKEDYILMSEPDHIIVKPIPNLARDGLGAAFPFFYIEPKKYETVLRKYFPKEKGPISNIDPIGNSPVIVGKEFLKKIAPTWMNVSLAMKKD

PETDKAFGWVLEMYAYAVASALHGVRNILHKDFMIQPPWDKEIGKTYIIHYTYGCDYTMKGELTYGKIGEWRFDKRSYDKVAPPKNLTLPPPGVPESVVTLVKMVNEATANIPNWWS

>GSVIVT00022269001

MIVRKSMGRASLLLLVLLALGFFFATYNLLTMIMHNRGSGLGNWMTDDFDVFDPITRMTKETKKVGNRNLYFHVALTATDAPYSQWQCRIMYYWYKKVKDMPGSEMGGFTRVLHSGSPDSLMDE

IPTFVVYPLPDGLDRGYIVLNRPWAFVQWLEKATIEEEYILMAEPDHIFVNPLPNLAHGGHPAGFPFFYIKPADNEKIIRKFYPKEKGPVTDVDPIGNSPVIIEKSQLEEIAPTWMNISLRMKD

DPETDKAFGWVLEMYAYAVASALHGVQHILRKDFMLQPPWDLEVGKKFIIHYTYGCDYNLKGELTYGKIGEWRFDKRSFLSGPPPKNLTLPPPGVPESVVRLVQMVNEATANIPGWDVE

>GSVIVT00030909001

MIVRKAMARISPLFLVVLSLGFFFATYNLLTMTIHNRATGTLVVDESDHRLLSDPIIEMPENVRKPKNAKLPFHIALTATDAPYSKWQCRIMYYWYKKKKDLPGSEMGGFTRILHSGSPDNLME

EIPTFVVDPLPAGLDRGYIVLNRPWAFVQWLEKATIEEEYILMAEPDHIFIKPLPNLAHGDYPAAYPFFYIKPVQNEKIIRKFYPEEHGPVTNVDPIGNSPVIIKRELLEKIAPTWMNVSLRMK

DDPETDKVFGWVLEMYAYAVASALHGVQHILQKDFMLQPPWDLETAKKFIIHYTYGCDYNLKGELTYGKIGEWRFDKRSYLRGPPPRNLSLPPPGVPESVVTLVKMVNEATANLPRWDTQ

>GSVIVT00032731001

MGCGNFFYVLLITFSVALITYNILISANAPLKQGFPGHPSSSSTPFSVDPIIKMPTDRSNPSSTKGKRLFHTAVTASDSVYNTWQCRVMYYWFKKFKDGPNSEMGGFTRILHSGKPDKFMHEIP

TFVAQPLPAGMDQGYIVLNRPWAFVQWLQQADIKEDYILMAEPDHIIVKPIPNLSRDGLGAAFPFFYIQPKQYESTLRKFYPEEKGPITNVDPIGNSPVIVGKESLKKIAPTWMNVSLAMKKDP

EADKTFGWVLEMYAYAVASALHDVGNILFKDFMIQPPWDTEIGKKFIIHYTYGCDYNMQGELTYGKIGEWRFDKRSFDSKWPPRNLPLPPPGVPESVVTLVKMVNEATANIPNWGS

>Cucsa086990

MIGRKTSPGFLVLLALGFLLASYNLITMSVHYKAPKGSWLAERAGKTNSKYHVAVTATDAPYSQWQCRIMYYWYKKVKDLPGSDMGSFTRVLHSGTPDNLMKEIPTFIVDPLPEGLDRGYVVLN

RPWAFVQWLEKANIEEEYILMAEPDHIFVKPLPNLAHGKNPAGFPFFYIKPADHEKIIRKFYPEENGPVTNIDPIGNSPVIIEKTLLEEIAPTWVNISLRMKDDPTTDKTFGWVLEMYAYAVAS

ALHGVRHTLRKDFMLQPPWDLEVGRNFIIHYTYGCDYTMKGELTYGKIGEWRFDKRTYLNGPPPRNLSLPPPGVPETVVRLVKMVNEATANIPDWGES

>Cucsa005190

MIGRKNTGQASPLFLVLLALTFCFVTYNLVTAIIQYGSVGREVGHDSYNHLSTDPIIEMPEKVKRKKTKSPFHVALTATDAPYSKWQCRIMYYWYKKKKNLPQSEMGGFTRILHSGKPDNLMDE

IPTMVVDPLPAGMDRGYIVLNRPWAFVQWLEKATIEEEYILMAEPDHIFVNPLPNLSDGGYPAAFPFFYIKPDQNHKILRKFFPEEYGPVNNIDPIGNSPVIIRKDLIEKIAPTWMNISLKMKE

DPEADKIFGWVLEMYAYAVASALHGVQHVLRKDFMLQPPWDLAIGRKFIIHYTYGCDYNLKGELTYGKIGEWRFDKRSHLRGPPPKNIPLPPRGVPESVITLVKMVNEATANLPNWEAT

>Cucsa162620

MGCGNLFFLVLVTFSVALITYNIILSANAPLKQELPGPSRSSSSITVDPVIKMPLDRSETSSSKRLFHTAVTASDSVYNTWQCRIMYYWFKKFKDGPNSEMGGFTRILHSGKPDKYMDEIPTFV

AQPLPAGMDRGYIVLNRPWAFVQWLQQADIKEDYILMSEPDHIIVKPIPNLSKDGLGAAFPFFYIEPKKYESQLRKFFPEDKGPITNIDPIGNSPVIVGKESLKKIAPTWMNVSLAMKKDPETD

KAFGWVLEMYAYAVASALHDVGNILYKDFMIQPPWDTEVGKKFIIHYTYGCDYDMKGKLTYGKIGEWRFDKRSYDNVVPPRNLPLPPPGVPESVVTLVKMVNEATANIPNWGS

>POPTR0001s28240

MIGRKNMGRASPLLLVLLALGFFFAMYNLLTLVIQYKDSSSGAGSGNPDPVIRMPTNLRKLGKSNLKFHVALTATDAPYSQWQCRIMYYWYKKMKNMPGSDMGKFTRVLHSGKGDHLMDEIPTF

VVDPLPEGLDRGYIVLNRPWAFVQWLEKATIEEEYILMAEPDHIFANPLPNLAHGDNPAGFPFFYIKPTEHEKIVQKFYPEEKGPVTDVDPIGNSPVIIKKSLLEEISPTWVNVSLRMKDDPET

DKAFGWVLEMYAYAVASALHGVRHILHEDFMLQPPWDLEVGKRFIIHYTYGCDYNMKGELTYGKIGEWRFDKRSYLSGPPPKNISLPPPGVPESVVRLVTMVNEATANIPGWDSLNSG

>POPTR0009s07430

MIGRKNMGRASPLLLVLLALGFFFATYNLLTLIIQYKDSSTGLGSGISDPVTGMPANSWKLGKSNLKFHVALTATDAPYSQWQCRVMYYWYKKMKSMPGSDMGKFTRVLHSGKGDHLMDEIPTF

IVDPLPDGLDRGYIVLNRPWAFVQWLEKATIEEDYILMAEPDHIFANPLPNLAHGDNPAGFPFFYIKPTEHEKIVRKFYPEEKGPVTDVDPIGNSPVIIKKSLLEEISPTWVNVSLRMKDDPET

DKAFGWVLEMYAYAVASALHGVRHILRKDFMLQPPWDLEVGKRFIIHYTYGCDYNMKGELTYGKIGEWRFDKRSYLSGPPPKNLTLPPPGVPESVVRLVKMVNEATANIPGWDSLNSG

>POPTR0001s06550

MGRASLLVLILLTIGFFFATYNLVAMIMHYRSIGKWAHDDSDGQIFLDPVTEMPEDVKKAKNAKMPFHVALTATDAPYSKWQCRIMYYWYKKKKDLPGSEMGGFTRILHSGKPDNLMDEIPTTV

VDPLPAGLDQGYVVLNRPWAFVQWLEKASIEEEYILMAEPDHVFINPLPNLARGGLPAAFPFFYIKPADHESIIRKYYPEEKGPVTNVDPIGNSPVIIKKELLEKIAPTWMNISLKMKRDQETD

KAFGWVLEMYAYAVASALHDVQHILRKDFMVQPPWDLATGKNFIIHYTYGCDYNLKGELTYGKIGEWRFDKRSYLGGPMPRNLALPPRGVPESVVTLVKMVNEATANIPNWDTE

>POPTR0003s19500

MGQASSPVLILLAFGFFFATYNLVTMTMHNRSIGKWVYDDSDGEAFFDPVIEMPEEVKKPKNARMPFHVALTATDAPYSKWQCRIMYYWYKKNRDLSGSEMGGFTRILHSGKPDNLMDEMPTVV

VDPLPAGLDRGYIVLNRPWAFVQWLEKTTIEEEYILMAEPDHILVNPLPNLARGGLPAAFPFFYIEPAKFENIVRKYYPEEKGPVTDIDPIGNSPVIIKKELLEKIAPKWMNVSLKMKNDKETD

KAFGWVLEMYAYAVAAALNDVQHVLRKDFMLQPPWDLSTRKFFIIHYTYGCDYNLKGQLTYGKIGEWRFDKRSYLRGPPPKNLPLPPPGVPESVVTLVKMVNEATANIPNWDAE

>POPTR0018s02160

MGCGNFFFTVLITLSVALITYNILISANAPLKQDLPGPSSRSTLLVDPVIKMPLERSRRSSFGKKRLFHTAVTASDSVYNTWQCRVMYYWYKKHKDGPNSEMGGFTRILHSGKPDKFMEEIPTF

IAQPLPAGMDQGYIVLNRPWAFVQWLQKTDIKEDYILMAEPDHIIVKPIPNLSKDGLGAAFPFFYIEPKKYESVLRKYFPEDKGPITNIDPIGNSPVIVGKESLKKIAPTWMNVSLAMKKDPET

DKAFGWVLEMYGYAVSSALHGVGNILYKDFMIQPPWDTEVGKKFIIHYTYGCDYDMKGKLTYGKIGEWRFDKRSYDTVIPPRNLPLPPPGVPESVVTLVKMVNEATANIPNWGS

>POPTR0006s27500

MGCGDLFFSLLITFSAALITYNILISANAPLKQELPGPSSRSSLLVDPIIKMPFGRSSFGKKRLFHTAVTASDSVYNTWQCRVMYYWYKKHKDGPNSEMGGFTRILHSGKPDKFMEEIPTFIAQ

PLPSGMDQGYIVLNRPWAFVQWLQKADIKEDYILMAEPDHIIVKPIPNLSKDGLGAAFPFFYIEPKKYESVLRKYFPEDKGPITTIDPIGNSPVIVGKESLKKIAPTWMNISLAMKKDPETDKA

FGWVLEMYAYAVSSALHGVGNILYKDFMIQPPWDTEIGKKFIIHYTYGCDYDMKGKLTYGKIGEWRFDKRSYDNVAPPRNLPLPPPGVPDSVVTLVKMVNEATSNIPNWGS

>AtHPAT3_At5g13500

MGKASGLLLFLLGFGFFVVTYNLLTLIVHNRSGVSNSDGSPLLDPVVQMPLNIRKAKSSPAPFHVALTATDAPYNKWQCRIMYYWYKQKKALPGSDMGGFTRILHSGNSDNLMDEIPTFVVDPL

PPGLDRGYVVLNRPWAFVQWLERATIKEDYVLMAEPDHVFVNPLPNLAVGGFPAAFPFFYITPEKYENIVRKYYPAEMGPVTNIDPIGNSPVIISKESLEKIAPTWMNVSLTMKNDPETDKAFG

WVLEMYGYAIASAIHGVRHILRKDFMLQPPWDLSTKGKFIIHYTYGCDYNMKGELTYGKIGEWRFDKRSHLRGPPPRNMSLPPPGVPESVVTLVKMVNEATATIPNWDTL

>AtHPAT1_At5g25265

MGCGGTLFYPLLITLSVALITYNIIISANAPLKQGFPGRSSSSDISIDPVIELPRGGGSRNNDGKRIRLFHTAVTASDSVYNTWQCRVMYYWFKKIQASAGPGSEMGGFTRILHSGKPDQYMDE

IPTFVAQPLPSGMDQGYVVLNRPWAFVQWLQQTDIKEDYILMSEPDHIIVKPIPNLAKDGLGAAFPFFYIEPKKYEKVLRKYYPEVRGPVTNIDPIGNSPVIVGKDALKKIAPTWMNVSLAMKK

DPEADKAFGWVLEMYAYAVSSALHGVSNILHKDFMIQPPWDIEVGDKYIIHYTYGCDYDMKGKLTYGKIGEWRFDKRSYDSKPPPRNLTMPPPGVSQSVVTLVKMINEATANIPNWGS

>AtHPAT2_At2g25260

MGFRGKYFFPILMTLSLFLIIRYNYIVSDDPPLRQELPGRRSASSGDDITYTVKTPSKKTKRLFHTAVTATDSVYSTWQCRVMYYWYNRFRDEPGSDMGGYTRILHSGRPDGLMDEIPTFVADP

LPSGVDKGYVVLNRPWAFVQWLQQAHIEEDYILMAEPDHIIVKPIPNLARGNLAAAFPFFYIEPKKYESVLRKFFPKENGPISRIDPIGNSPVIVTKNALMKIAPTWMNVSLAMKNDPQTDKAF

GWVLEMYAYAVSSALHGVSNILHKDFMIQPPWDTETKKTFIIHYTYGCDFDMKGKMMVGKIGEWRFDKRSYGDKPPPRNLTLPPRGVPESVVTLVTMINEATANIPNWES

>Os01g16600

MNAAARKAAAAARLPAAVLVVVAVGAFLISYNLLAIVLRGGGGAATGAGRERDPVVAMPGWMRAAGSAGGGRRRPFHVALTATDAAYSRWQCRVMYYWYKRMQARPEGADMGGFTRVLHSGKPD

ALMGEIPTFVVDPLPAGKDHGYVVLNRPWAFVQWLEKAKIEEEYILMAEPDHIFVRPLPNLARDD-

PAAFPFFYITPSEHESVLRKYYPKERGPVTNIDPIGNSPVIIKKIQLEKIAPTWMNVSIQMKEDQETDKAFGWVLEMYAYAVASALHGVQHILRKDFMIQPPFDTKLGNTFIIHFTYGCDYTLK

GVLTYGKIGEWRFDKRAYQDRPPPRNLTLPPPGVPESVVTLVKMVNEATANLPGWDDGR

>Os05g32060

MSGRKNAGKASPFLLLLISVGCFFATYNFLTMVGHGRSRDAGPRKILGGVGGVGGGGSDPSKRFHVALTATDALYSQWQSRVMYYWYREMRDRPGSDMGGFTRILHSGKPDGLMDEIPTLVVDP

LPEGADRGYIVLNRPWAFVQWLKKSNIKEDYVLMAEPDHIFVRPLPNLAHGDEPAAFPFFYIKPTENEIILRKFFPEENGPVSKIDPIGNSPVIIKKAQLEKIAPTWMNISLKMKEDVETDKAF

GWVLEMYAYAVASALHGVHYSLRKDFMIQPPWDAKSDNTFIIHYTYGCDYTLKGELTYGKIGEWRFDKRSYLRSPPPRNLTLPPPGVPESVATLVKMVNEATANIPGWDEER

>Os06g08180

MAAPCRGSRGALPLLLISLSAAYLTYTALLSSRSLLPLPTASFPGATASRRLASGRPTAAAAFHTAVTASGSLYNTWQCRVMYYWFKRAREAGGGGGAEMGGFTRILHSGKPDAFVDEIPTFVA

DPLPAGTDQGYVVLNRPWAFVQWLQKADIQEEYILMAEPDHLIVKPIPNLSRDGRSAAFPFFYIEPKKYENVLRKFFPEHEGPITKIDPIGNSPVIARKESLARIAPTWMNISIAMKKDPETDK

AFGWVLEMYAYAVASALHGVGNILHKEFMIQPPWDLEIGDAFIIHYTYGCDYDMKGKLTYGKIGEWRFDKRSYDSKPPPRNLPLPPNGVPQSVVTLVKMVNEATANIPNWDSYAAA

>GRMZM2G035595

MDAAVRKAAGAGIARAPALVLALVAAGAFLISYNFFTMLFHGGGGIGAAVTAGTRDPVVAMPAWMRAAADTEARRRPFHVALTATDAPYSRWQCRVMYFWYKRMQARPGGEAMGGFTRVLHSGK

PDGLMDEIPTFVVDPLPAGKDHGYVVLNRPWAFVQWLQKAKIEEEYILMAEPDHIFVKPLPNLAHDDDPAAFPFFYITPSEHEKIIRKYYAKERGPVTDIDPIGNSPVIIKKTILEKIAPTWMN

VSIQMKEDEETDKVFGWVLEMYAYAVASALHGVHHILRKDFMIQPPFDTKLQNTFIIHFTYGCDYSLKGELTYGKVGEWRFDKRSFPDRPPPRNLTLPPPGVPESVVTLVKMVNEASANLPRWD

DGI

>GRMZM2G055020

MSGRKNAGKVSPWLLVLICLGCFFVTYNLLTMPSRGRDGPRKFLGGGGDRHSTASLGSGSDSDPAKRFHVALTATDALYSQWQSRIMHYWYKEMRDRPGSDMGGFTRILHSGKPDGLMDEIPTM

VVDPLPEGKDQGYIVLNRPWAFVQWIQKAKIVEEYILMAEPDHVFVKPLPNLSHGDEPAAFPFFYIKPTENEKILRKFFPEEKGPISNIDPIGNSPVIIKKAQLEKIAPTWMNVSLKMKEDQET

DKAFGWVLEMYAYAVASALHGVHHSLHKDFMIQPPWDLKTDNTFIIHYTYGCDYSMKGQLTYGKIGEWRFDKRSYLQSPPPRNLSLPPPGVPESVVTLVKMVNEATANIPGWEDDR

>GRMZM2G072550

MAAPCGRGGGTLTLVLVALSAAFLT-

YNVLISFHSSLQPLPSSFPTASRRFGAAGSARRRAFHTAVTASGSAYNTWQCRVMYHWFKEARRAPGGDEMGGFTRILHSGKPDEFVDEIPTFVADPLPDG-

DQGYIVLNRPWAFVQWLQKADIKEDYILMAEPDHIIVKPIPNLSRDGQAAAFPFFYIEPKKYENVLRKFFPEDKGPITKIDPIGNSPVIIEKESLGRIAPTWMNVSLAMKKDPDADKSFGWVLE

MYAYAVASALHGVGNILRKDFMIQPPWDLEVGDSFIIHYTYGCDYDMTGKLTYGKIGEWRFDKRSYTDKPPPRNLPLPPHGVAQSVVTLVKMVNEATANIPNWDSYAAAF

>Sb03g010840

MNAAAAAAARKAAGGGMARAPALVLALVAAGAFLISYNFFAMLFRGGGGGIGGAAASSGTRDPVVAMPAWMREAADTEARRR-

PFHVALTATDAPYSRWQCRVMYFWYKRMQARPGGEAMGGFTRVLHSGKPDGLMDEIPTFVVDPLPAGKDHGYVVLNRPWAFVQWLQKAKIEEEYILMAEPDHIFVKPLPNLAHDDDPAAFPFFY

ITPSEHEQIIRKYYAKERGPVTDIDPIGNSPVIIKKTLLEKIAPTWMNVSIQMKEDEETDKIFGWVLEMYAYAVASALHGVQHILRKDFMIQPPFDTKLGNTFIIHFTYGCDYSLKGELTYGKV

GEWRFDKRSFPDGPPPRNFTLPPPGVPESVVTLVKMVNEASANLPRWDDGI

>Sb09g019030

MSGRKNAGKVSPWLLGLISLGCFFVTYNLLTMHGRGRDGPRKFLGGGEDHDSTGSGSDPAKRFHVALTATDALYSQWQSRIMHYWYKEMRDRPGSDMGGFTRILHSGKPDGLMDEIPTMVVDPL

PEGKDKGYIVLNRPWAFVQWIQRAKIVEDYILMAEPDHVFVKPLPNLAHGDEPAAFPFFYIKPTDNEKILRKFFPEEKGPVSNIDPIGNSPVIIKKAQLEKIAPTWMNVSLKMKEDQETDKAFG

WVLEMYAYAVASALHGVRHSLRKDFMIQPPWDLKTDNTFIIHYTYGCDYSMKGQLTYGKIGEWRFDKRSYLQSPPPRNLSLPPPGVPESVVTLVKMVNEATANIPGWEDER

>Sb10g005440

MAAPCGRGTLTLFLVSLSAAFLTYNVLVSFRSSLQPLPSSFPTASSSSSSSSSSSSRRFGAARRRAFHTAVTASGNAYNTWQCRVMYHWFKEARRAPGGAEMGGFTRVLHSGKPDEFMDEIPTF

VADPLPDG-

DQGYIVLNRPWAFVQWLQKADIKEDYILMAEPDHIIVKPIPNLSRDGQAAAFHFFYIEPEKYENVLRKFFPEDKGPITKIDPIGNSPVIIEKESLGRIAPTWMNVSIAMKNDPDADKSFGWVLE

MYAYAVASALHGVGNILHKEFMIQPPWDLEVGDAFIIHYTYGCDYDMKGKSTYGKIGEWRFDKRSYDDKPPPRNLPLPPNGVPQSVVTLVKMVNEATGSIPNWDSYAASY

>Sm235499

MALSRIFLLLCLFLVASVLTYQSLSVARSSVVRNFGRSRAAGAAS-

SALVSMPRDFAKARRRERRFHTAVTATSALYNRWQCRIMYYWYKKFKDEAGSEMGGFTRVLHSGKPDNLMDEIPTFVVDPLPPGMDKGYVVLNRPWAFVQWLQKANIEEEYIFMAEPDHVILRP

IPNLAIDDMPAAYPFFYITPKSHEKLLRRYFPEDRGPITNIDPIGNSPVIIKKSHLLRVAPTWANVSLQLKNDPAADKAFGWVLEMYGYAVASALHGIQHILHKNFMVQPPWDGKLGEVYMIHY

TYGCDYTMKGELTYGKVGEWRFDKRSYTQKVMPQNLTLPPPGVPETVVRLVQMVNEATANLPNWTMQ

>Sm150302

MGRATFPLLILITVALFIATHNTVSMVFKHRGEMSDQKAMSDMDGNPAGGVDTYDPLIKMPRSFKNSKKKKKRMFHTVMTASSVPYNNWQSRVMYYWYKQQKEKAGSEMGGFTRLLHTGKADHL

MDEIPTYVVQPLPEGTDKGFVVLNRPWAFVQWFRDVEIEEDYVFMAEPDHIIIRPIPNLSTGELPAAFPFFYIDPKQFQKTLRRWYPESKGPITNIDPIGNSPVIIKKSLLKEIAPTWMNVSLE

MKNDPQADKDFGWVLEMYGYAVASAMHGVQHVLRKDFMLQPPYDTKLEKKFIIHYTYGCDYTLKGEPMFGKFGEWRFDKRSYGQGAPPRNLTMPPKGVHESVVTLVRMINEATANIPNWKEGER

>Sm231983

MGRISFFFLVLLFVAFTAAIYNTARFIAANDSTLGGGEEFDFNPGGKKKLFHTVVTSSSGSYARWQCRIMYYWFKKFKDQPGSEMGGFTRILHSAAPDDLMDEIPTFVADPLPSGLDKGYIVLH

RPWAFVQWLRNATIEEDYIFMAEPDHLITKPLPNLASRLRPAAFPFFYITPEKYKREVGKFFKGPVQAIDPVGNSPAIIHKAQLLRIAPTWHNLSIQMKQDQETDKAFGWVLEMYAYAIASAVH

GVKHTLRKDFMIQPPWDTSLGDKYIIHYTYGCDYTLEGVLTYGKVGEWRFDKRSFTAGAPPRNLTLPPANVPGSVAMLVKMINQATADIPNWKAGE

>22688338_peptide|Brapa_Chifu-401_v1.2|Bra020495|Bra020495

MGCGGTTLFYPLLITLSVALITYNIIISSNTPLKQGFPSSSSSSSSIIDPVIELPRGGSRIRGNNERKRRLFHTAVTASDSVYNTWQCRVMYYWFKKIRASAGPGSEMGGFTRILHNGKPDQYM

DEPTFVAQPLPPGTDQGYVVLNRPWAFVQWLQQADIKEDYILMSEPDHLIVKPIPNLAKDGYGAAFPFFYIEPKKYEKVLRKYYPEERGPVTDIDPIGNSPVIVGKEALKKIAPTWMNVSLAMK

KDPEADKAFGWVLEMYAYAVSSALHGVSNILHKDFMIQPPWDTEVGDKYIIHYTYGCDYDMKGKLTYGKVGEWRFDKRSYDSTPPPRNLTMPPPGVSQSVVTLVKMVNEATANIPNWGE*

>22693428_peptide|Brapa_Chifu-401_v1.2|Bra008825|Bra008825

MGKASSLLLFLLSFGFFVVTYNLLTLILHNRSGLSNSDGSSPLLDPIVQMPHRNSQTSSAPFHVALTATDAPYNKWQCRIMYYWYKQKKALPGSDMGGFTRILHSGNTDNLMDEIPTFVVDPLP

PGDQGYVVLNRPWAFVQWLERATIIEDYVLMAEPDHVFVNPLPNLAVGGHPAAFPFFYITPEKFENIVRKYYPVEMGPVSNIDPIGNSPVIISKESLEKIAPTWMNVSLTMKNDPDTDKAFGWV

LEMYGYAVASALHGVRHILHKDFMIQPPWDLSTKGKFIIHYTYGCDYNMQGELTYGKIGEWRFDKRSHLRGPPPRNISMPPRGVPESVVTFVKMVNEATSNIPNWDTL*

>22711645_peptide|Brapa_Chifu-401_v1.2|Bra006204|Bra006204

MGKASSLLLFLLGFGFFVVTYNLMTLIVHNRSGSVSSPLLDPVVQMPNRKAKTSSPAPFHVALTATDAPYNKWQCRIMYYWYKQKKALPGSDMGGFTRILHSGNRDNLMDEIPTFVVDPLPPGL

DRYVVLNRPWAFVQWLERATIEEDYVLMAEPDHVFVNPLPNLAVGGYPAAFPFFYITPEKYENIVRKYYPVEMGPVSNIDPIGNSPVIISKESLENIAPTWMNVSLTMKNDPDTDKAFGWVLEM

YGYAVASALHGVRHILRKDFMLQPPWDLSTKGKFIIHYTYGCDYNMKGELTYGKIGEWRFDKRSHLRGPPPRNISMPPPGVPESVVTLVKMVNEATANIPNWETL*

>22717275_peptide|Brapa_Chifu-401_v1.2|Bra036538|Bra036538

MGCGGTLFYPLLITLSVALITYNILISSNAPLKQGFPSSSSYEDPVIQLPRGGSRIRGNNDKKIRLFHTAVTASDSVYNTWQCRVMYYWFKKAKASAGPGSEMGGFTRILHNGKPDKYMDEIPT

FVQPLPSGMDQGYVVLNRPWAFVQWLQQTDIKEDYVLMSEPDHVIVKPIPNLAKEGFGAAFPFFYIEPKKYEKVLRKYYPEERGPVTNIDPIGNSPVIVGKEALKKIAPTWMNVSLAMKKDPEA

DKAFGWVLEMYAYAVSSALHGVRNVLHKDFMIQPPWDKEVGDKYIIHYTYGCDYDMKGHLTYGKKGEWRFDKRSYIKSPPPKNLTMPPPGVPQSVVTLVKMVNEATANLPDWGS*

>22719643_peptide|Brapa_Chifu-401_v1.2|Bra032030|Bra032030

MYYWYNRFRDEPGSEMGGYTRILHSGRPDGLMNEIPTFVANPLPSGVDQGYVVLNRPWAFVQWLQQAHIEEDYILMAEPDHIIVKPIPNLARGNLGAAFPFSYIEPKKYEAVLRKFFPKDNGLI

SKDPIGNSPVIVSKNALMKIAPTWMNVSLAMKNDPQTDKAFGWVLEMYAYAVSSALHGVSNILHKDFMIQPPWDTESKNTYIIHYTYGCDFDMKGKMMVGKIGEWRFDKRSYGDKPPPRKLTLP

PQGVPESVVTLVSMVNEATANIPNWES*

>Cpa:evm.model.supercontig_157.63|evm.model.supercontig_157.63|evm.TU.supercontig_157.63

MGRVSPLLLVLLALGFSFATYNLLTMIMHNKAFNSARWAVDDAELSDQMAGERTLGNSNSKFHVALTATDAPYSQWQCRIMYYWYKKVRDMPGSEMGKFTRVLHSGNPDNLMEEIPTFVVDPLP

EGLDRGYIVLNRPWAFVQWLEKATIEEEYILMAEPDHIFVNPLPNLAQGNRPAGFPFFYIKPAEHEQIIRKFYPEERGPVTDIDPIGNSPVIIKKSILMDISPTWLNISLRMKDDPATDKAFGW

VLEMYAYAVASALHGVQHTLRKDFMLQPPWDLEVGKKFIIHYTYGCDYNLKAELTYGKIGEWRFDKRSYLSGPPPRNLSLPPPGVPESVVRLVTMVNEATANIPRWDTLNHG*

>Cpa:evm.model.supercontig_187.19|evm.model.supercontig_187.19|evm.TU.supercontig_187.19

MACGNLFYPLLITFSVALITYNIILAANAPLKQELPGPSKSSSIYVDPVIKMPMDRSKLYGSGSRKRLFHTAVTASNSVYNTWQCRIMYYWFKKFKNGPNSEMGAFTRILHSGKPDNFMDEIPT

FVQPLPAGTDQGYIVLNRPWAFVQWLQQADIKEDYILMAEPDHIIVKPIPNLSKDGLGAAFPFFYIEPKKYESVLRKFFPEDRGPITNIDPIGNSPVIIGKDSLKKIAPTWMNVSLAMKKDSEA

DKAFGWVLEMYAYAVSSALHGVGNILYKDFMIQPPWDTEVGKKFIIHYTYGCDYDMKGRLTYGKIGEWRFDKRSYDNTVPPRNLPLPPPGVPESVVTLVKMVNEATANIPNWG*

>Cpa:evm.model.supercontig_403.4|evm.model.supercontig_403.4|evm.TU.supercontig_403.4

MGQASSLLLVLLAFGFFIVTYNLLTMLMHNRAVGKWISDDSDGGIFFDPVVKMPENVKNPKNAKLPFHVALTATDAPYSKWQCRIMYYWYKKKKDLPGSDMGGFTRILHSGNPDNLMDEIPTVV

VDLPSGLDRGYIVLNRPWAFVQWLEKATIEEEYILMAEPDHIFISPLPNLARGGYPAAFPFFYIKPEKNEQIIRKFYPEENGPVTNVDPIGNSPVIIKKDLLEKIAPTWMNVSLKMKNDPETDK

AFGWVLEMYAYAVASALHGVQHILRKDFMLQPPWDLETAKKFIIHYTYGCDYNMKGELTYGKIGEWRFDKRSYLRGPPPRNLSLPPPGVPESVVTLVKMVNEATANIPNWDTQ*

>Gra:Gorai.010G216000.1|Gorai.010G216000.1|Gorai.010G216000

MISRKNMGSASPLLLITLVLGVCFATYNLVTMVMHNRSISKLTIYNSDGGIFVDPIIEMPESVRKLKRAKMPFHVALTATDAPYSKWQCRIMYYWYKKQKDLPGSEMGGFTRILHSGNPDNLMN

EITVIVDPLPAGLDRGYIVLNRPWAFVQWLEKATIEEEYVLMAEPDHIFVTPLPNLAHGGYPAAFPFFYIKPDQNEKLLRKFFPEEMGPVTNIDPIGNSPVIIKKELLEKIAPTWMNVSLKMKD

DPETDKTFGWVLEMYAYAVASALHGVQHVLQKDFMLQPPWDLEIGKKYIIHYTYGCDYNMKGELTYGKIGEWRFDKRSFLRGPPPRNLPLPPPGVPESVVTLVKMVNEATANIPNWDAE*

>Gra:Gorai.011G215700.1|Gorai.011G215700.1|Gorai.011G215700

MIVRKNMGRASPLVLVTLVLGFCFATYNLVTMIMHSRAISKWVEDDANGGIFFDPVIKMPENVRKPKNARLPFHVAVTATDATYSQWQCRIMYYWYKKKKDLPGSDMGGFTRILHSGSPDNFMD

EIPTIVVDPLPEGLDRGYIVLNRPWAFVQWLEKATIEEDYILMAEPDHIFINPLPNLASGGFPAAFPFFYIKPADYGKVLRKFFPEEMGPVTNIDPIGNSPVIIKKDLLKKIAPTWMNVSLKMK

DDPETDEAFGWVLEMYAYAVGSALHGVQHILRKDFMLQPPWDFEIGKKFIIHYTYGCDYNMKGELMYGEIGEWRFDKRSFLRGPPPRNLSLPPPGVPESVVTLVKMVNEATANIPNWEA*

>Gra:Gorai.001G035500.1|Gorai.001G035500.1|Gorai.001G035500

MGCGNVFFTLIITLSVALITYNILISANASLKQELPGPSTSSIIDPIIQMPVEKSRKYGSNAEKRLFHTAVTASDSVYNTWQCRVMYYWFKKHKNGPNSDMGGFTRILHSGKPDNYMNEIPTFI

ARPLPAGMDQGYIVLNRPWAFVQWLQKADIKEDYILMAEPDHIIVKPIPNLSKDGLGAAFPFFYIEPKKYELVLRKYFPEEKGPITNIDPIGNSPVIVGKDSLKKIAPTWMNVSLAMKKDPETD

KAFGWVLEMYAYAVSSALHGVGNILYKDFMIQPPWDTEIGNKFIIHYTYGCDYNLKGKLTYGKIGEWRFDKRSFDTEAPPRNLPLPPPGVPESVVILVKMVNEATSNIPNWGS*

>Gra:Gorai.006G124300.1|Gorai.006G124300.1|Gorai.006G124300

MKRVSPLFLVLLVLGFFFATYNLLTMIIHYKTSTSEQWDLSNPFIQMPGNLMEGEGSNSKYHVALTATDAPYSQWQCRIMYYWYKKVKEMPGSDMGKFTRILHSGKPDNLMEEIPTFVVDPLPE

GLDRGYIVLNRPWAFVQWLEKAMIDEEYILMAEPDHVFVNPLPNLARGKHPAGFPFFYIKPSDHKHVIRKYYPEEKGPVTNIDPIGNSPVIIKKSVLEKIAPTWMNISLRMKDDPVTDKAFGWV

LEMYAYAVASALHDVHHILHKDFMLQPPWDTEVGKKFIIHYTYGCDYNLKGELTYGKIGEWRFDKRSYLSGPPPRNLSLPPPGVPESVVRLVKMVNEATEDIPNWDTLNRG*

>Egr:Eucgr.C01703.1|Eucgr.C01703.1|Eucgr.C01703|synonym:Egrandis_v1_0.016833m

MGCGNTFFSVLITFSVALITYNILISANAPLKQELPGPSDPSSGLSVDPVIKMPLDRSRRFGGGGGGGKRLFHTAVTASDSVYNTWQCRVMYYWFKKHQNGPNSEMGGFTRILHSGKPDAYMDE

IPFVAQPLPSGMDQGYIVLNRPWAFVQWLQQADIKEDYILMSEPDHVIVKPIPNLSRDGLGAAFPFFYIEPKKYETVLRKFFPEEKGPITNIDPIGNSPVIVGKESLKKIAPTWMNVSLAMKKD

PETDKAFGWVLEMYAYAVSSAFHGVGNILYKDFMIQPPWDKELGEKFIIHYTYGCDYNMKGELTYGKIGEWRFDKRSYDKVPPPKYLPLPPPGVPESVVTLVKMVNEATANIPNWGS*

>Egr:Eucgr.G03228.1|Eucgr.G03228.1|Eucgr.G03228|synonym:Egrandis_v1_0.016719m

MFGMKAMRRASPVLLFLLAALFCFATYNILAMMRHNRAIRNFVGANTRSVLESDPIVEMPEHVKKPKHVKSPFHIALTATAAPYSKWQCRIMYYWYKQKKDLPGSDMGGFTRILHSGSPDNLMD

EIPTMVVDPLPAGLDRGYVVLNRPWAFVQWLEKAAIEEEYILMAEPDHIFIRPLPNLAHERFPAAFPFFYIKPEQNEKIVRKFYPEEHGPVTNIDPIGNSPVIIRKDLLGKIAPTWMNVSLRMK

QDPETDKTFGWVLEMYAYAVASALHGVQHILRKDFMLQPPWDLETGKRFIIHYTYGCDYNLKGELTYGKIGEWRFDKRSHLRGPPPRNLSLPPPGVPESVVTLVKMVNEATANIPNWDTS*

>Egr:Eucgr.J00694.1|Eucgr.J00694.1|Eucgr.J00694|synonym:Egrandis_v1_0.017286m

MPTIERKHVRRVSPLFVVFGLVFLFAAYNLLHMVMHNKGPNPGAAVTDALGMSDPVTGLRYHVAVTATDAPYSQWQCRIMYYWYKKVRDMPGSDMGNFTRILHSGRTDHLMEEMHTFVVDPLPD

GLDRGYIVLNRPWAFVQWLESATIEEEYILMAEPDHIFVSPFPNLAKGSHPAGYPFFYIKPAEKAKIIRKFYPKEKGPLTDIDPIGNSPVIIQKSILEEIAPTWMNVSLRMKEDPETDKAFGWV

LEMYGYAVASALHKVKHILRKDFMLQPPWDLEVGKSFIIHYTYGCDYSMKGELTYGKIGEWRFDKRSYLSGPPPRNLSLPPPGVPESVVQLVKMVNEATANIPKWETLNRS*

>Egr:Eucgr.J01177.1|Eucgr.J01177.1|Eucgr.J01177|synonym:Egrandis_v1_0.016698m

MTGRRNTGSASVPLLVLLSLGFCFATYNLQTIIWHNIAIMRSAVDDSNRKLLSDPILEMPDDLQKQKTSGSPFHVALTATDAPYSKWQSRIMYYWYKKKKDLPGSEMGGFTRILHSGNRDNLMD

EIPTIVVDPLPAGLDRGYIVLNRPWAFVQWLEKATIEEEYILMAEPDHIFVNPLPNLADGGYPAAFPFFYIKPAENENIIRKFFPQENGPVTNVDPIGNSPVIIKTDLLKKIAPTWMNVSLRMK

DDSETDKAFGWVLEMYAYAVASALHGVRHVLRKDLMLQPPWDLQTAKKFIIHYTYGCDYNLKGELTYGKIGEWRFDKRSYLRGPPPRNLSLPPPGVPESVVTLVKMVNEATANIPNWDTE*

The first 41 sequences including *Lotus japonicus* (Lj), *Medicago truncatula* (Mt), soybean (*Glycine max*, Gm), grape (*Vitus vinifera*, GSVIVT), cucumber (*Cucumus sativus*, Cucsa), poplar (*Populus trichocarpa*, POPTR), and *Arabidopsis thaliana* (*At*), rice (*Oryza sativa*, Os), sorghum (*Sorghum bicolor*, Sb), and maize (*Zea mays*, GRMZM), *Selaginella moellendorfii* (Sm) are completely the same as in the study of RDN1 (Schnabel et al., 2011). The latter 16 sequences with longer headings are obtained from a BLAST search for the Phytozome website (http://www.phytozome.net/) containing *Brassica rapa* Chiifu-401 v1.2, papaya (*Carica papaya* ASGPB v0.4), cotton (*Gossypium raimondii* v2.1), and eucalyptus (*Eucalyptus grandis* v2.0).

Appendix S2. The deletion series of PLENTY proteins used in this study

```
                                          30                             60                             90
                                           *                              *                              *
   1 ATGATTGTGAGGAAAAACATGGGAAGGGCAAAGTCACTTCTTATGCTGCTCATGGTGCTTGGGTTTTTCTTCGCCACTTATAATCTGGTG
     M  I  V  R  K  N  M  G  R  A  K  S  L  L  M  L  L  M  V  L  G  F  F  F  A  T  Y  N  L  V
                       >>>>>>>>>>>>>>>>>>>>>>
                       PLENTY_CDS_F

                                         120                            150                            180
                                           *                              *                              *
  91 TCTATGATCATGGACCATAGGGCTGGAAATTGGGTTGCAGATGGGTTGGAATCTTTTGATAGAAAAATGTTGGGTTCGGCAAGCACTAAT
     S  M  I  M  D  H  R  A  G  N  W  V  A  D  G  L  E  S  F  D  R  K  M  L  G  S  A  S  T  N
        >>>>>>>>>>>>>>>>>>                                              >>>>>>>>>>>>>>>>>>>>
         ⊿N2_PLENTY_inf_F                                            ⊿N1_PLENTY_CDS_F, ⊿N1_PLENTY_inf_F
                                                                                      <<<<<<<<<<<
                                                                                      N_PLENTY_CDS_R

                                         210                            240
                                           *                              *
 181 GCGAAATACCATGTTGCCCTGACTGCAACTGATGCTGCTTACAGCCAATGGCAATGCCGGATCATGTACTATTGGTATAAGAA
     A  K  Y  H  V  A  L  T  A  T  D  A  A  Y  S  Q  W  Q  C  R  I  M  Y  Y  W  Y  K  K
     <<<<<<<<<<<<
     N_PLENTY_CDS_R

                                        1020                           1050                           1080
                                           *                              *                              *
 991 CCTCCTGGAGTTCCTGAAAGTGTGGTGCGGCTTGTAAAGATGGTCAATGAGGCCACTGCAAACATACCTGAATGGGATTCATTAAATAGA
     P  P  G  V  P  E  S  V  V  R  L  V  K  M  V  N  E  A  T  A  N  I  P  E  W  D  S  L  N  R
                                                                    <<<<<<<<<<<<<<<<<<<<<<<
                                                                    PLENTY_CDS_R

1081 AGCTGA
     S  *
     <<<<<<
     PLENTY_CDS_R
```

Nucleotides and amino acid coding sequence sequences of PLENTY are shown. The two N-terminal deleted PLENTY constructs (ΔN2-PLENTY and ΔN1-PLENTY) and the PLENTY construct containing only the N-terminal region (N-GFP) are shown in red lines, orange lines, and blue lines, respectively. The hybridized region of the primers used to construction of the respective partial CDS series (Supplementary Table S2) are shown as 5’ >>> 3’ with respective colors. Amino acids highlighted in yellow were predicted as transmembrane domain by TMHMM 2.0 also predicted in MtRDN1 (Schnabel *et al.*, 2011).
